# Molecular detection of *Toxoplasma gondii* in ready-to-eat salad mixes: multi-country survey using a validated and harmonised standard operating procedure, Europe, 2021 to 2022

**DOI:** 10.2807/1560-7917.ES.2025.30.22.2400594

**Published:** 2025-06-05

**Authors:** Rafael Calero-Bernal, Martha Betson, Iva Slana, Barbora Bartosova, Gianluca Marucci, Alessia Possenti, Gema Álvarez-García, Nadja Bier, Anne Mayer-Scholl, Rebecca P Berg, Umer Chaudhry, Nadia M López-Ureña, Weronika Piotrowska, Jacek Sroka, Gro S Johannessen, Rebecca Davidson, Filip Dámek, Radu Blaga, Sandra Thoumire, Barbora Zalewská, Helga C Waap, Pikka Jokelainen, Marco Lalle

**Affiliations:** 1 Animal Health and Zoonoses (SALUVET) Research Group, Animal Health Department, Faculty of Veterinary Sciences, Complutense University of Madrid, Madrid, Spain; 2 Department of Comparative Biomedical Sciences, School of Veterinary Medicine, University of Surrey, Guildford, the United Kingdom; 3 Department of Microbiology and Antimicrobial Resistance, Veterinary Research Institute, Brno, Czechia; 4 Department of Infectious Diseases, Istituto Superiore di Sanità, Rome, Italy; 5 Department of Biological Safety, Unit Diagnostics, Pathogen Characterization, Parasites in Food, German Federal Institute for Risk Assessment (BfR), Berlin, Germany; 6 Laboratory of Parasitology, Department of Bacteria, Parasites & Fungi, Statens Serum Institut, Copenhagen, Denmark; 7 Lewyt College of Veterinary Medicine, Long Island University New York, New York, the United States; 8 Microtech S.R.L. (Laboratory), Moca, Espaillat, Dominican Republic; 9 Department of Parasitology and Invasive Diseases, Bee Diseases and Aquatic Animal Diseases, National Veterinary Research Institute, Pulawy, Poland; 10 Section for Food Safety and Animal Health Research, Norwegian Veterinary Institute, Tromsø, Norway; 11 Section for Food Safety and Animal Health Research, Norwegian Veterinary Institute, Ås, Norway; 12 Anses, INRAE, Ecole Nationale Vétérinaire d’Alfort, Laboratoire de Santé Animale, BIPAR, Maisons-Alfort, France; 13 University of Agricultural Sciences and Veterinary Medicine, Cluj-Napoca, Romania; 14 Laboratory of Parasitology, The National Institute for Agricultural and Veterinary Research (INIAV), Oeiras, Portugal; 15 Secretariat for Infectious Disease Preparedness and One Health, Statens Serum Institut, Copenhagen, Denmark

**Keywords:** Leafy greens, ready-to-eat salads, oocysts, Toxoplasma gondii, harmonisation, ring-trial, Europe

## Abstract

**Background:**

Most *Toxoplasma gondii* infections in humans are considered foodborne, but the relative importance of the various routes of infection is largely unknown. Consumption of green produce contaminated with *T. gondii* oocysts has been identified as a possible source.

**Aim:**

We aimed to estimate the occurrence and prevalence of *T. gondii* oocysts in commercially available ready-to-eat (RTE) salad mixes in 10 European countries.

**Methods:**

A real-time PCR-based method for oocyst detection was developed and optimised by two laboratories and validated in an interlaboratory test. This detection method and a harmonised sampling strategy were applied in a multi-country study. Multivariable logistic regression was used to investigate risk factors for oocyst contamination of RTE salad.

**Results:**

The real-time PCR method had a detection limit of 10 oocysts per 30 g of salad. We collected 3,329 RTE salad samples (baby leaf and cut leaf mixes) from October 2021 to September 2022. The prevalence of *T. gondii* oocyst contamination was 4.1% (95% confidence interval (CI): 3.4–4.8%; n = 3,293). In multivariable regression analysis, winter season, sampling and packaging of salad in Northern Europe and production of salad in Western Europe were associated with detection of *T. gondii*, with no statistically significant differences between salad types.

**Conclusion:**

We estimated the prevalence of *T. gondii* oocysts in RTE leafy green salads using a validated and standardised procedure to assess the potential risk for human infection; highlighting the need to address this risk at each critical point of the salad production chain.

**Key public health message**

**What did you want to address in this study and why?**

The parasite *Toxoplasma gondii* can cause severe disease in humans. People can acquire the parasite by eating raw or undercooked infected meat or unwashed fruits or vegetables contaminated with the parasite. We wanted to investigate *T. gondii* in commercial ready-to eat (RTE) salads in European countries to estimate the importance of these food products as sources of *T. gondii*.

**What have we learnt from this study?**

Using a molecular method, we detected *T. gondii* in 4.1% of 3,293 RTE salad samples from 10 European countries. Sampling in winter season, sampling and packaging of salad in Northern Europe and salad grown in Western Europe were associated with detection of *T. gondii* from salad samples.

**What are the implications of your findings for public health?**

As RTE salads are intended to be consumed raw without further processing, *T. gondii* in salad from European countries may lead to infections in humans. Measures to prevent and control contamination of salads are necessary. Further washing of RTE salads might be recommended for specific risk groups such as pregnant people.

## Introduction

*Toxoplasma gondii* is an intracellular zoonotic protist that can infect a wide variety of warm-blooded animals [1]. *Toxoplasma gondii* has a complex life cycle, where felids act as definitive hosts, and almost all warm-blooded animals, including humans, may serve as intermediate hosts. Definitive hosts become infected through ingestion of viable tissue cysts containing bradyzoites. The parasite then multiplies in the small intestine, leading to the formation of environmentally resistant oocysts shed with faeces. Oocysts may contaminate water and food, serving as a source of infection for intermediate hosts (responsible for 6–17% of human infections). Carnivorous and omnivorous intermediate hosts may also be infected through ingestion of infected animal tissue, for example raw or undercooked meat (responsible for 30–60% of human infections) [2,3]. Oocyst and bradyzoite ingestion are followed by systemic multiplication of the fast-replicating stage called tachyzoites and may result in systemic toxoplasmosis. When infection reaches the chronic stage, tissue cysts are formed [1].

Infection with *T. gondii* in humans may go unnoticed or be associated with mild clinical signs (e.g. fever, lymphadenopathy) [1]. However, particularly persons with a weakened immune system may develop severe neurological and respiratory symptoms, which may result in death. In addition, infections during pregnancy can cause congenital toxoplasmosis (CT) leading to abortions, fetal malformations and malignancies in the nervous system [4]. It is estimated that one third of humans worldwide have been exposed to *T. gondii* [5]. Differences in the prevalence of toxoplasmosis in European countries explained ca 23% of between-country variability in mortality and age-standardised disability-adjusted life years (DALYs) [6]. In 2022, the incidence of CT in the European Union/European Economic Area (EU/EEA) countries was 5.8 cases per 100,000 live births [7]. Congenital toxoplasmosis is one of the diseases with highest disease burden in the EU/EEA countries (21st position) [8]. Therefore, there is a need to quantify the relative contribution of various transmission routes for *T. gondii* to enable design of appropriate intervention strategies [5,9,10]. In a recent systematic review, the overall detection rate of *T. gondii* oocysts ranged from 0.09% to 100% in soil, water, fresh produce and bivalve molluscs [9].

In surveys performed in European countries including Czechia, Italy, Portugal, Spain and Switzerland using PCR-based methods, the occurrence of *T. gondii* oocysts in fresh produce matrices (e.g. fruit, leafy greens or other vegetables) varied from 6.0% to 13.6% (reviewed by [4]). In recent years, an increase in human toxoplasmosis cases associated with consumption of fresh produce has been observed [3]. Few studies have investigated ready-to-eat (RTE) salad mixes, but in one Italian study using a specific real-time PCR with amplicon sequencing as a confirmatory test, 0.8% of 648 RTE salads samples tested were potentially contaminated with *T. gondii* oocysts [11]. The risk of acquiring *T. gondii* via salad consumption is unknown and comprehensive risk assessments on, for example, parasite load and oocyst viability, are needed [9]. Further investigations are justified because: (i) consumption of RTE salads is rapidly growing [12]; (ii) there is no mandatory surveillance for foodborne parasites in fresh produce; and (iii) the currently used methods have inconsistencies and limitations [9,13]. We aimed at developing a molecular-based method and estimating the occurrence and prevalence of *T. gondii* oocysts in RTE salads at retail in Europe.

## Methods

### Study design

To ensure collection of reliable and comparable data on *T. gondii* contamination of RTE salads, a step-by-step approach was undertaken (Figure A). We selected a molecular-based detection method and RTE matrices tested based on previously conducted literature reviews [9,13]. This was followed by assessment, implementation and validation of a standard operating procedure (SOP) (Figure A and B), and the application of the SOP in a field survey conducted in 10 European countries (Figure A and C).

**Figure:**
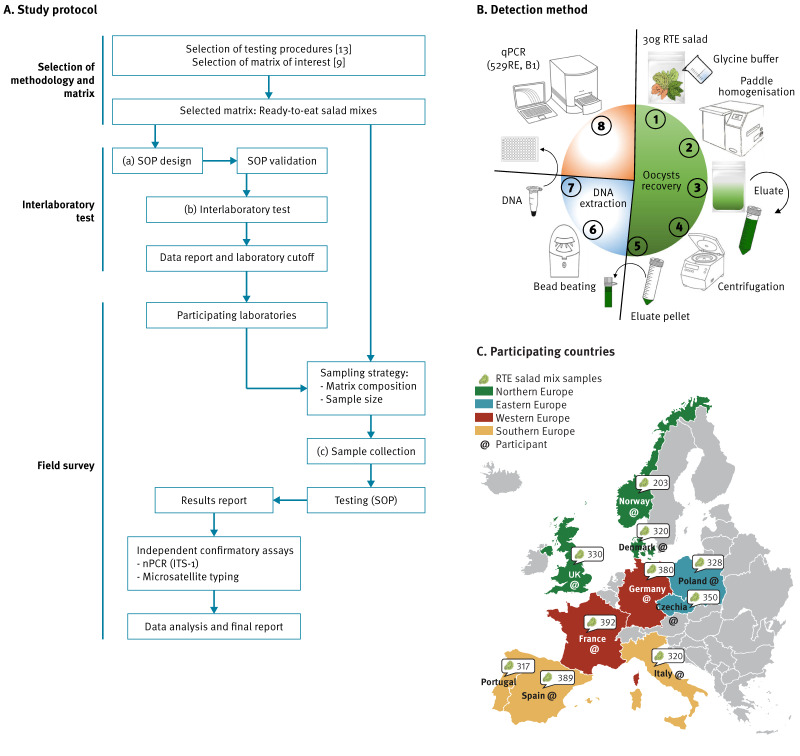
Overview of a survey for detection of *Toxoplasma gondii* in ready-to-eat salad mixes, Europe, 2021–2022

ITS: internal transcribed spacer; RTE: ready-to-eat; SOP: standard operating procedure; UK: United Kingdom; UN: United Nations.

Panel A. First, we selected testing procedures and the matrix, followed by design and validation of the SOP (a) with an interlaboratory test (b). Next, we designed sampling strategy (c) for a field survey of RTE salads in retail stores in 10 European countries.

Panel B. SOP was validated by Istituto Superiore di Sanità, Italy and Veterinary Research Institute, Czechia.

Panel C. Countries participating in the field survey are coloured by the geographical region according to the UN classification. Southern Europe: Italy, Portugal, Spain; Western Europe: France and Germany; Northern Europe: Denmark, Norway, the UK; Eastern Europe: Czechia and Poland. Laboratories participating in the interlaboratory test are indicated by @.

### Assessment of the performance of the standard operating procedure for detection of *Toxoplasma gondii* in ready-to eat salads

Development and assessment of the SOP was initially performed by Istituto Superiore di Sanità, Rome, Italy and Veterinary Research Institute, Brno, Czechia. The SOP consisted of three steps: *T. gondii* oocyst recovery, DNA extraction and detection by real-time PCR (Figure B). The complete SOP is available in Supplementary File 1. For the experimental work, *T. gondii* oocysts of the Savannah isolate (type II) isolated from faeces of experimentally infected cats [14], were further purified by sucrose density gradient flotation [15]. Oocysts (6 × 10^7^/mL) were stored at 4°C in sterile phosphate-buffered saline (PBS) supplemented with 1 × antibiotic/antimycotic solution (Euroclone, Pero, Italy).

The genomic DNA was extracted from 1 × 10^7^ oocysts using the FastDNA SPIN Kit for Soil (MP Biomedicals, Santa Ana, the United States (US)) according to the manufacturer’s protocol and eluted in 100 µL of water. Commercially available RTE mixed salads (containing different amounts of green and red baby lettuce, arugula, baby spinach, roman baby lettuce) were purchased from local stores (Italy and Czechia) and used as a matrix. Briefly, for *T. gondii* oocyst recovery [16], 30 g of RTE salad was transferred into a filter bag with 200 mL of washing buffer (glycine 1 M, pH 5.5) and the sample was mixed twice using a paddle blender. The eluate was collected into 50 mL conical tubes and centrifuged at 2,500 × g for 10 min at 4°C. Supernatants were discarded, pellets (here termed salad sediment) were combined, washed once with 50 mL of molecular grade water and transferred into a 2 mL vial.

The mixed salads or salad sediments were artificially spiked with *T. gondii* oocysts (100, 50 or 10 oocysts). Ten oocysts was the minimum number of oocysts reproducibly counted under microscope after serial dilution of oocyst master stock solution (data not shown). Procedures for preparation of oocyst aliquots for spiking and salad leaf and salad sediment spiking are available in Supplementary Methods. From the salad sediments, DNA was extracted using either the QIAamp Fast DNA Stool Mini Kit (Qiagen, Hilden, Germany), FastDNA SPIN Kit for Soil (MP Biomedicals) or DNeasy PowerSoil Kit (Qiagen) as described in the SOP in Supplementary File 1. Multiplex real-time PCR amplifications were performed in 96-well plate by targeting the B1 multicopy gene and the 529 bp repetitive element (529RE) from the genomic DNA of *T. gondii* and a recombinant internal amplification control (IAC) [17]. Plasmids with a cloned single copy of B1 gene, 529RE and IAC have been previously described [17]. Real-time PCR reaction conditions were as follows: activation for 5 min at 95°C, one cycle; denaturation for 15 s at 95°C (ramping rate 4.4) followed by annealing/amplification for 40 s at 60°C (ramping rate 2.2), 45 cycles. Four different qPCR master mixes were compared using two platforms: SensiFAST Probe No-ROX (Meridian Life Science-Academic, London, the United Kingdom (UK)) and QuantiFast Multiplex PCR Kit (Qiagen) polymerases on a LightCycler96 (Roche, Basel, Switzerland) thermocycler, Lightcycler 480 Probes Master (Roche) or Luna Universal Probe qPCR Master Mix (New England Biolabs, Ipswich, US) polymerases on LightCycler 480 Probes Master thermocycler. Samples were considered positive if at least one real-time PCR replicate was positive for any of the targets (B1 or 529RE) with quantification cycle (Cq) ≤ 39.0. The Cq threshold was positioned just above the background amplification signal. The sensitivity (SE) of the method [18] was defined as the percentage of positive samples at each spiking level (number of positive samples/total spiked samples). The percentage of positive real-time PCR replicates at any genomic DNA (gDNA) concentration (oocyst equivalent) was expressed as the probability of detection (POD) (number of positive real-time PCR replicates/total number of real-time PCR replicates) [18]. The limit of detection (LOD_95_) was the lowest gDNA concentration (or oocyst number equivalent) level for which the POD was > 95% (100% in our study).

### Interlaboratory validation of the standard operating procedure

Prior to participating in the interlaboratory validation (ILV), the SOP and video tutorials were sent to nine participating laboratories. The videos are available in Supplementary Videos 1, 2 and 3. Four weeks before the distribution of the ILV material, each participant received detailed information on how to conduct the ILV, available in Supplementary File 2. The participants were provided with three panels of samples. Panel 1, aiming at evaluating the overall performance from oocyst recovery to real-time PCR, consisted of five vials, containing (or not) enumerated *T. gondii* (Savannah isolate, type II) oocyst suspension in 100 µL sterile PBS (supplemented with 1 × antibiotic/antimycotic solution, A5955, Merck, Darmstadt, Germany), for spiking of leafy salads. Participants were instructed to buy their own RTE salad for spiking; preferably 200 g of the same brand and batch of green baby lettuce monotype, and to record details on the form provided. If *T. gondii* was detected in nonspiked samples, this was considered in evaluating the recovery efficiency in the spiked samples. Panel 2, aiming at evaluating the efficiency of DNA extraction, was composed of five vials containing salad sediment spiked, or not, with *T. gondii* oocyst suspension. Salad sediment was prepared from 1 kg of commercial RTE mix as described above and aliquots (150 µL) of pooled pellets were prepared corresponding to 30 g of leafy salad. Pellets were stored at −20°C until use. Absence of detectable *T. gondii* contamination was verified by DNA extraction from randomly selected sediment aliquots (n = 3) using FastDNA SPIN Kit for Soil (MP Biomedicals) and multiplex real-time PCR. Just before shipping, sediments were thawed and individually spiked with sterile water with or without *T. gondii* oocysts and sealed. Panel 3, aiming at verifying the efficiency of real-time PCR, consisted of three vials with pooled genomic DNA (40 µL) from control salad sediments (n = 6) or sediments spiked (n = 6) with *T. gondii* oocysts (10 or 100 oocysts) extracted using FastDNA SPIN Kit for Soil (MP Biomedicals) according to the SOP. In addition, a positive control gDNA (2.8 ng/µL = 5,000 oocysts/µL) obtained from in vitro culture of *T. gondii* RH strain tachyzoites [16] was provided to each participant to prepare the standard curve. A stock of IAC (5 × 10^8^ copies/µL in TE buffer) was also provided. All tubes of each panel set were plugged and sealed with plastic paraffin film, individually identified with a code and vacuum sealed in a plastic bag. Each panel set was placed inside a polystyrene carton with an adequate number of ice packs to guarantee the maintenance of an internal temperature 4–15°C during the transport. Insulating material was added to avoid undesired shaking of package contents. Samples were shipped using an international courier.

### Sampling strategy and data collection

Two types of RTE products were chosen for the field survey: baby leaf mixes and cut salad mixes. Details on the RTE salad composition are provided in Supplementary Methods. The salads were collected in retail stores in 10 countries (Figure C), in the same or a nearby city of the participating laboratory, thus limiting sample transport time and costs. Samples were collected weekly, with a mean of 7–8 samples per week, corresponding to 320 samples per country, from October 2021 to September 2022. The overall target number of samples was 3,200, with 400 samples of cut salad and 400 of baby leaves per season, based on an estimated prevalence of *T. gondii* oocyst contamination of 1–20%. The power to detect medium and large effect sizes was estimated to > 90% [9]. The estimates are summarised in Supplementary Table S1. To improve representativeness, at least two different brands of each type of salad mix were sampled. Collected metadata included, but were not limited to, the country where the salad was purchased (sampling country), organic production (organic Y/N), farming country (country of origin), processing and packaging country (packaging country), packaging date and use-by date.

### Sample testing procedures for field survey

Samples were collected and processed for oocyst recovery before the use-by date of the product and transported from retail stores to laboratory under refrigerated conditions (e.g. using plastic bags with cool packs). The samples were stored at 4°C until analysis, and 30 g of RTE salad samples were tested according to the SOP, available in Supplementary File 1. After the oocyst recovery step, the salad sediment was either processed immediately or stored at −20°C until DNA extraction. The vegetable pellet suspension was thawed, and DNA was then extracted using FastDNA Prep SPIN Kit for Soil (MP Biomedicals), a bead-beating based DNA extraction kit and eluted with 100 µL of reagent grade water. More details are available in Supplementary Table S2.

For each DNA extraction kit with a different batch number, a positive control for the extraction, consisting of salad sediment obtained from the test portion and spiked with 50 *T. gondii* oocysts (Savannah isolate, type II), was processed in parallel. Multiplex real-time PCR amplification was performed according to the SOP. Each sample was run in triplicate with a positive control for DNA extraction, a reference genomic DNA of *T. gondii* (positive control) and water (negative control). A standard curve of a 10-fold serial dilution of quantified reference *T. gondii* genomic DNA was run during each experiment. All the anonymised raw data of the field study are available in Supplementary Excel Table S1.

### Statistical analyses

Statistical analyses were carried out using Stata version 14.2 (https://www.stata.com/). *Toxoplasma gondii* oocyst contamination was classified as a binary variable (positive/negative). We used the exact method (Clopper-Pearson binomial confidence intervals) for calculation of 95% confidence intervals (CI). Sampling months were categorised into seasons: autumn (October–December), winter (January–March), spring (April–June) and summer (July–September). Countries of origin, packaging and sampling were categorised by European region according to the United Nations Statistics Division (UNSD) classification [19]. For region of origin and packaging, Eastern Europe included Czechia, Hungary and Poland, Southern Europe included Italy, Portugal and Spain, Western Europe included France and Germany and Northern Europe included Denmark, Norway, Sweden and the UK. For sampling region, the categorisation was the same, except for Eastern Europe which did not include Hungary, and Northern Europe did not include Sweden. We performed univariable analysis using the Fisher’s exact test to identify risk factors associated with *T. gondii* oocyst contamination, considering sampling season, salad type (cut salad, baby leaves, mixed), production system (conventional or organic), farming, packaging and sampling region. Variables showing a statistical association (p < 0.05) with *T. gondii* oocyst contamination (all variables apart from salad type and production system) were included stepwise in multivariable logistic regression models. Likelihood ratio tests were used to compare the models. Models with ≥ 3 explanatory variables performed less well than models with two variables. Therefore, three separate models each were included to present associations with region of origin, packaging and sampling. The unknown categories for the region of origin and packaging region variables were omitted from the relevant models.

### *Toxoplasma gondii* nested PCR and genotyping assay

Samples yielding positive amplification for one or both targets (B1 and/or 529RE) were shipped frozen to the Veterinary Faculty of the Complutense University of Madrid, Spain for confirmation. Samples were tested undiluted by single-tube nested PCR targeting the internal transcribed spacer 1 (ITS-1) ribosomal region [20] in a Veriti Thermal Cycler (Applied Biosystems, Waltham, US); amplicons of expected size (227 bp) were visualised in 1.5% (w/v) agarose gel electrophoresis and further subjected to both strand Sanger sequencing using internal amplification primers in an ABI 3730 DNA Analyzer (Applied Biosystems) at the Center for Genomic Technologies of the Complutense University of Madrid. Sequences obtained were edited using BioEdit software, version 7.0.5.3 (https://bioedit.software.informer.com). Alignments were carried out with the Clustal Omega Software (https://www.ebi.ac.uk/jdispatcher/msa/clustalo) and consensus sequences were compared with available sequences from the National Center for Biotechnology Information (NCBI) database using the basic local alignment search tool (BLAST) (http://blast.ncbi.nlm.nih.gov/Blast.cgi). Attempts were then made to genotype samples yielding strong amplification bands (ca 10 ng/µL DNA) using microsatellite (MS) fragment analysis following a recently harmonised protocol [21].

## Results

### Performance of the standard operating procedure

The performance was initially evaluated in a two-laboratory comparative study using plasmids individually harbouring a single copy of *T. gondii*-specific markers, the B1 gene or 529 repetitive element (529RE), or an internal amplification control (IAC). The amplification efficiency (Cq values comparison) for both *T. gondii* markers was highly comparable and only dependent on target copy number. Nevertheless, Cq values were dependent on the thermocycler but independent of the DNA polymerase used. The Cq values obtained for the triplex real-time PCR using plasmid DNA, different DNA polymerases and real-time PCR platforms are available in Supplementary Table S3. Since the copy numbers of B1 and 529RE in the *T. gondii* genome differ (30–35 copies of B1; 200–300 copies of 529RE) [22,23], DNA extracted from a known number of *T. gondii* oocysts was used for further evaluations. At any *T. gondii* gDNA concentration tested, Cq values depended on the thermocycler but were less dependent on the polymerase used. The LOD_95_ for the real-time PCR was established at 0.1 oocyst equivalent/reaction (0.056 fg of *T. gondii* gDNA) (POD = 100%) for both molecular targets, both thermocyclers and for three of four DNA polymerases used. The Cq values obtained for the triplex real-time PCR using DNA extracted from oocysts, different DNA polymerases and real-time PCR platforms are available in Supplementary Table S4. Since no specific enrichment procedure (e.g. immunocapture) is currently available for *T. gondii* oocysts, detection of *T. gondii* oocysts in a complex matrix relies on a combination of efficient DNA extraction and sensitive endpoint molecular assay. To evaluate the most efficient DNA extraction procedure, two commercial bead-beating based kits (FastDNA Prep SPIN Kit for Soil, MP Biomedicals and QIAamp Fast DNA Stool Mini Kit, Qiagen), relying on mechanical disruption of the oocyst, were compared against a DNA extraction kit relying on freeze/thaw and chemical lysis of the oocysts (DNeasy Power Soil Kit, Qiagen). The mixed salads or salad sediments were spiked with different amounts of *T. gondii* oocysts and processed for DNA extraction and real-time PCR. The Cq values obtained for the triplex real-time PCR using DNA from spiked salad sediment, different DNA polymerases and real-time PCR platforms are available in Supplementary Table S5. For each spiking dose, Cq values and standard deviations for both targets were consistently lower using the bead-beating based protocols compared with the freeze/thaw and chemical lysis. The LOD_95_ for the entire procedure using the bead-beating based protocol corresponded with the lowest spiking dose (10 oocysts) with a POD and sensitivity of 100% for the 529RE target, independently of DNA polymerase and thermocycler used. Although LOD_95_ for the B1 marker was 10 oocysts, POD and sensitivity were markedly lower than 100% for one polymerase used.

### Interlaboratory validation of the standard operating procedure

To strengthen the validation of the SOP, an interlaboratory validation (ILV) was organised among nine laboratories (Figure C). Details of the reagents and equipment used, real-time PCR results and SE and POD are provided in Supplementary Excel Table S2. All participants correctly identified the positive and negative samples (Panel 3). Variability of Cq values (ΔCq > 3), included in the standard curves, demonstrated that differences in the reagents and the equipment affect the amplification performance making the assay suitable for detection but not for quantification of the oocysts. Six laboratories had an LOD_50_ (instead of LOD_95_) of 10 oocysts/30 g of salad (Panel 1), corresponding to a sensitivity of 78% for both molecular targets, with POD being 63% and 76% for B1 and 529RE. One participant underperformed in the oocyst recovery step (high Cq values and low number of positive replicates from spiked salad and/or salad sediment) whereas two laboratories had problems with the DNA extraction step. To ensure comparable performance in the RTE salad survey, these three laboratories received additional reference material (i.e. vials containing, or not, counted *T. gondii* oocysts suspension to be spiked onto RTE salad) from the ILV organiser. Bead-beating time was identified as the limiting cause in *T. gondii* oocyst DNA extraction. Following this optimisation of bead-beating time, all nine laboratories were able to correctly apply the SOP thus reaching the 10 oocysts/30 g of salad as LOD (data not shown).

### Ready-to-eat salad survey

A total of 3,329 samples were collected and subjected to oocyst recovery step. Samples were equally distributed between the two main categories of RTE mixes (baby leaves and cut salad), between countries and seasons (Table 1). According to information on the salad packages, the region of origin and packaging country or region, e.g. EU could be assigned to 2,360 (70.9%) and 3,210 (96.4%) samples, respectively. Reliable real-time PCR results were obtained from 3,293 samples (98.9%). Data from 36 samples were excluded from analysis due to concerns about cross-contamination during sample processing or absence of amplification of the IAC, indicative of PCR inhibition. Of the 3,293 samples, 135 (4.1%; 95% CI: 3.4–4.8%) were positive for at least one target gene (Table 2): 69 (51.1%) were positive for 529RE, 24 (17.8%) for B1 and 42 (31.1%) for both target genes.

**Table 1:** Ready-to-eat salad samples collected in a survey of *Toxoplasma gondii* in fresh produce, Europe, 2021–2022 (n = 3,329)

Category	Samples collected (n)	%	95% CI
Sampling country			
Czechia	350	10.5	9.5–11.6
Denmark	320	9.6	8.6–10.7
France	392	11.8	10.7–12.9
Germany	380	11.4	10.4–12.5
Italy	320	9.6	8.6–10.7
Norway	203	6.1	5.3–7.0
Poland	328	9.9	8.9–10.9
Portugal	317	9.5	8.5–10.6
Spain	389	11.7	10.6–12.8
United Kingdom	330	9.9	8.9–11.0
Salad type			
Baby leaves	1,618	48.6	46.8–50.3
Cut salad	1,691	50.8	49.1–52.5
Mixed	16	0.5	0.3–0.7
Unknown	4	0.1	0.03–0.3
Sampling season^a^			
Autumn	776	23.3	21.9–24.8
Winter	949	28.5	27.0–30.1
Spring	874	26.3	24.8–27.8
Summer	730	21.9	20.5–23.4
Region of origin^b^			
Eastern Europe	399	12.0	10.9–13.1
Southern Europe	1,244	37.4	35.7–39.0
Western Europe	99	3.0	2.4–3.6
Northern Europe	51	15.3	11.4–20.1
Europe	562	16.9	15.6–18.2
Europe/Morocco	5	0.2	0.05–0.4
Unknown	969	29.1	27.6–30.7
Packaging country			
Czechia	35	1.1	0.7–1.5
Denmark	292	8.8	7.8–9.8
France	494	14.8	13.6–16.1
Germany	290	8.7	7.8–9.7
Hungary	58	1.7	1.3–2.2
Italy	414	12.4	11.3–13.6
Norway	203	6.1	5.3–7.0
Poland	324	9.7	8.7–11.0
Portugal	192	5.8	5.0–6.6
Spain	390	11.7	10.6–12.9
Sweden	28	0.8	0.6–1.2
UK	330	9.9	8.9–11.0
EU	160	4.8	4.1–5.6
Unknown	119	3.6	3.0–4.3
Organic production			
No	3,044	91.4	90.4–92.4
Yes	285	8.6	7.6–9.6

CI: confidence interval; EU: European Union; UK: United Kingdom.

^a^ Autumn (October–December), winter (January–March), spring (April–June) and summer (July–September).

^b^ Eastern Europe: Czechia, Hungary and Poland; Southern Europe: Italy, Portugal and Spain; Western Europe: France and Germany; Northern Europe: Denmark, Norway, Sweden and the UK.

**Table 2:** Ready-to-eat salad samples tested for *Toxoplasma gondii*, Europe, 2021–2022 (n = 3,293)

Category	Samples tested (n)	Real-time PCR positive				ITS-1 PCR	
		n	%	95% CI	2 markers	Tested (n)	Positive (n)
Sampling country							
Czechia	350	0	0.0	0–1.0	0	0	NA
Denmark	320	12	3.8	2.0–6.5	2	12	1
France	392	24	6.1	4.0–9.0	1	24	2
Germany	374	8	2.1	0.9–4.2	1	8	0
Italy	317	6	1.9	0.7–4.1	1	6	1
Norway	202	1	0.5	0.01–2.7	0	1	0
Poland	327	9	2.8	1.3–5.2	3	9	1
Portugal	316	10	3.2	1.5–5.7	2	10	3
Spain	389	16	4.1	2.4–6.6	4	16	10
UK	306	49	16.0	12.1–20.6	28	47	4
Salad type							
Baby leaves	1,600	60	3.8	2.9–4.8	19	58	7
Cut salad	1,673	75	4.5	3.5–5.6	23	75	15
Mixed	16	0	0.0	NA	0	0	NA
Unknown	4	0	0.0	NA	0	0	NA
Season^a^							
Autumn	758	11	1.5	0.7–2.6	3	10	2
Winter	943	59	6.3	4.8–8.0	24	59	6
Spring	866	38	4.4	3.1–6.0	11	37	7
Summer	726	27	3.7	2.5–5.4	4	27	7
Region of origin^b^							
Eastern Europe	398	9	2.3	1.0–4.2	3	9	1
Southern Europe	1,241	44	3.5	2.6–4.7	8	44	15
Western Europe	99	7	7.1	2.9–14.0	0	7	1
Northern Europe	51	1	2.0	0.05–10.4	0	1	0
Europe	561	9	1.6	0.7–3.0	1	9	1
Europe/Morocco	5	0	0.0	NA	0	0	NA
Unknown	938	65	6.9	5.4–8.7	30	63	4
Packaging country							
Czechia	35	0	0.0	NA	0	0	NA
Denmark	292	11	3.8	1.9–6.6	2	11	1
France	493	26	5.3	3.5–7.6	1	26	2
Germany	285	6	2.1	0.8–4.5	1	6	0
Hungary	58	0	0.0	NA	0	6	1
Italy	411	6	1.5	0.5–31.5	1	0	-
Norway	202	1	0.5	0.01–2.7	0	1	0
Poland	323	9	2.8	1.3–5.2	3	9	1
Portugal	192	8	4.2	1.8–8.0	2	8	2
Spain	390	16	4.1	2.4–6.6	4	16	10
Sweden	28	1	3.6	0.1-18.3	0	1	0
UK	306	49	16.0	12.1–20.6	28	47	4
EU	160	0	0.0	0–2.3	0	0	-
Unknown	118	2	1.7	0.2–6.0	0	2	1
Organic production							
No	3,008	127	4.2	3.5–5.0	41	125	21
Yes	285	8	2.8	1.2–5.4	1	8	1
Total	3,293	135	4.1	3.4–4.8	42	133	22

CI: confidence interval; EU: European Union; ITS: internal transcribed spacer; UK: United Kingdom.

^a^ Autumn (October–December), winter (January–March), spring (April–June) and summer (July–September).

^b^ Eastern Europe: Czechia, Hungary and Poland; Southern Europe: Italy, Portugal and Spain; Western Europe: France and Germany; Northern Europe: Denmark, Norway, Sweden and the UK.

Most positive results were from samples collected in winter (n = 59; 6.3%; 95% CI: 4.8–8.0) (Table 2). Compared with the autumn, contamination with *T. gondii* was higher in winter (model 1: odds ratio (OR) = 4.79; 95% CI: 2.49–9.21; model 2: OR = 4.93; 95% CI: 2.56–9.49 and model 3: OR = 5.03, 95% CI: 1.94–13.04), in spring (model 1: OR = 3.29, 95% CI: 1.66–6.50 and model 2: OR = 3.52; 95% CI: 1.78–6.95) and summer (model 1: OR = 2.63; 95% CI: 1.29–5.35 and model 2: OR = 2.72; 95% CI: 1.34–5.54) (Table 3). The number of *T. gondii* positive samples detected per season per country is presented in Supplementary Figure S1.

**Table 3:** Multivariable regression analysis of factors associated with detection of *Toxoplasma gondii* in ready-to-eat salad samples, Europe, 2021–2022 (n = 3,293)

Category	Number	OR	95% CI	p value
Model 1 (n = 3,293)				
Season^a^				
Autumn	758	Reference		
Winter	943	4.79	2.49–9.21	< 0.0001
Spring	866	3.29	1.66–6.50	0.001
Summer	726	2.63	1.29–5.35	0.008
Sampling region^b^				
Eastern Europe	677	Reference		
Southern Europe	1,022	2.16	1.02–4.57	0.043
Western Europe	766	3.29	1.56–6.96	< 0.0001
Northern Europe	828	6.00	2.95–12.18	< 0.0001
Model 2 (n = 3,015)				
Season				
Autumn	734	Reference		
Winter	840	4.93	2.56–9.49	< 0.0001
Spring	765	3.52	1.78–6.95	< 0.0001
Summer	676	2.72	1.34–5.54	0.006
Packaging region^c^				
Eastern Europe	416	Reference		
Southern Europe	993	1.31	0.62–2.80	0.479
Western Europe	778	1.95	0.92–4.14	0.082
Northern Europe	828	3.61	1.77–7.36	< 0.0001
Model 3 (n = 2,350)				
Season				
Autumn	496	Reference		
Winter	692	5.03	1.94–13.04	< 0.001
Spring	673	2.68	0.99–7.28	0.053
Summer	489	2.45	0.85–7.05	0.097
Region of origin^c^				
Eastern Europe	398	Reference		
Southern Europe	1,241	1.43	0.69–3.00	0.337
Western Europe	99	3.86	1.34–11.16	0.013
Northern Europe	51	1.06	0.13–8.70	0.957
Europe	561	0.66	0.26–1.68	0.385

CI: confidence interval; OR: odds ratio.

^a^ Autumn (October–December), winter (January–March), spring (April–June) and summer (July–September).

^b^ Eastern Europe: Czechia and Poland; Southern Europe: Italy, Portugal and Spain; Western Europe: France and Germany; Northern Europe: Denmark, Norway, Sweden and the UK.

^c^ Samples with an unknown region and samples from EU/Morocco were not included in the model.

Almost 28.5% of samples had no information of the region of origin, while 37.5% originated from Southern Europe (Italy, Portugal and Spain) (Table 2). Samples originating from Western Europe had the highest percentage of positives (7.1%; 95% CI: 2.9–14.0), but there was no significant difference in the positivity between regions. Most positive samples were from the packaging countries the UK (16%; 95% CI: 12.1–20.6), France (5.3%; 95% CI: 3.5–7.6), Portugal (4.2%; 95% CI: 1.8–8.0), Spain (4.1%; 95% CI: 2.4–6.6) and Denmark (3.8%; 95% CI: 1.9–6.6). These results largely mirror the results per sampling country, with the UK reporting most positives (16%; 95% CI: 12.1–20.6), followed by France (6.1%; 95% CI: 4.0–9.0), Spain (4.1%; 95% CI: 2.4–6.6) and Denmark (3.8%; 95% CI: 2.0–6.5) (Table 2). In the multivariable regression analysis, compared with Eastern Europe, there was a greater risk of *T. gondii* contamination in salad sampled in Northern Europe (OR = 6.00, 95% CI: 2.95–12.18), Southern Europe (OR = 2.16, 95% CI: 1.02–4.57) and Western Europe (OR = 3.29, 95% CI: 1.56–6.96) (Table 3). Salad packaged in Northern Europe was more likely to be contaminated with *T. gondii* than salad packaged in Eastern Europe (OR = 3.61; 95% CI: 1.77–7.36). Samples from salad farmed in Western Europe (Table 3) were more likely to be positive for *T*. *gondii* than salad farmed in Eastern Europe (OR = 3.86; 95% CI: 1.34–11.16). However, results should be interpreted with caution since information regarding region of packaging and/or origin was limited.

### Confirmatory assays by nested PCR

To provide sequence-based evidence for presence of *T. gondii* in positive samples, we tested 133 (98.5%) of 135 samples. Two samples could not be included because of low numbers of the DNA available. The nPCR amplification was successful for 39 (29.3%) samples and Sanger sequencing for 22 amplicons with sequences showing 100% homology with the ITS-1 fragment. Multiple alignment of the sequenced fragments is shown in Supplementary Figure S2. We attempted to genotype nine *T. gondii*-positive samples, but none of them resulted in reliable amplification of markers (data not shown).

## Discussion

By using a harmonised sampling and detection method, we detected *T. gondii* DNA in 4.1% (95% CI: 3.4–4.8%) of 3,293 RTE salad samples. These salads were sold in nine of the 10 European countries surveyed, thus pointing to the potential contamination by *T. gondii* oocysts. The need to develop a harmonised method for detection of *T. gondii* and application of similar sampling strategies has been highlighted by multiple authors [9,13,24] to assess the risk of *T. gondii* oocysts in fresh produce. Several surveys, using various detection methods, have investigated the presence of *T. gondii* oocysts in fresh produce (reviewed by [9]). In the previous studies, different oocyst concentration methods and other approaches including microscopy, molecular-based methods or bioassays have been used for oocyst detection. Molecular methods are preferrable due to the high throughput of testing and their anticipated high sensitivity and specificity [9,13]. However, appropriate validation data are essential for robust results [13]. For instance, in one study, *Eimeria papillata* was targeted as a surrogate for coccidia of public health concern in leafy greens and berry fruits [25]. This method was further adapted for *T. gondii* and subsequently used in a nation-wide survey on leafy greens collected in retail outlets in 11 cities in Canada [26].

The detection method we used had some limitations. We did not quantify oocysts or confirm the presence of oocysts visually or test them for viability and infectivity. For further quantitative risk characterisation, this information is necessary. However, considering that our method had a LOD of 10 oocysts per 30 g of salad and one salad portion per person is ca 80–100 g according to the European Commission food-based dietary guidelines for vegetables [27], the infection risk for a consumer should not be considered negligible.

Our study results are in agreement with previous studies from Czechia, Italy, Portugal, Spain and Switzerland. Using PCR-based methods, prevalence of *T. gondii* oocysts in different fresh produce products (e.g. fruit, leafy greens or other vegetables, including RTE salads) ranged from 6.0 to 37.2% in these studies [9,28]. However, a direct comparison of our results with other studies is difficult due to differences in sample preparation and analytical methods [13]. In one Italian study, 0.8% of the 648 tested RTE salad mixes potentially contained *T. gondii* oocysts [11], which is within the 95% CI: 0.7–4.1 of the results of the Italian samples in our study.

We amplified the B1 gene and/or 529 RE. As expected, 529 RE was a more sensitive target due to the higher number of copies compared with the B1 gene. We are aware that the set of instruments and reagents applied in the present study, including the Cq value established as cutoff, could lead to discrepancies between participating laboratories. However, the LOD was ca 10 oocysts per 30 g of sample, high Cq values for both targets generally obtained in positive samples by all laboratories might be indicative of a low oocyst load/number presence in these specimens. In another study, 62–554 oocysts per gram of vegetable product were estimated [11].

Several factors may have affected the observed differences in the prevalence of *T. gondii* contamination between countries. Unfortunately, information on country of origin was not always available on salad packages. Differences in the manufacturing practices or RTE production workflows may have had an impact [29]. A systematic study aimed at identifying the risks at each step of the production chain is warranted to understand the higher prevalence in certain countries, for example the UK.

Unfortunately, we did not succeed in genotyping *T. gondii,* possibly due to the limited sensitivity of the MS multiplex PCR [21]. Information on the genotypes of *T. gondii* in environmental samples is scarce in Europe [30]. Genotyping environmental samples is challenging as parasites are in low concentrations [21].

As *T. gondii* DNA was detected in commercially available RTE salad mixes in many European countries, we need to better understand the production systems to assess the associated risks and implement prevention and control measures. This is particularly relevant since RTE salads are intended to be consumed raw without any further treatment by the consumer [31]. Information about the packaging facility was available in only a few countries (Denmark, France, Germany and Italy), which demonstrates the absence of harmonised labelling of salad packaging in Europe, thus hampering tracing of the point of contamination. Starting from 1 January 2025, the Commission Delegated Regulation (EU) 2023/2429 is applicable in all EU countries improving labelling on salad packages (including country of origin) to ensure product traceability [32]. Environmental factors that may contribute to the contamination of leafy greens are numerous and well-known [24,33]. In our study, production type (organic/conventional) and season were included. Given the limited information on the package labelling, only season was significantly associated with detection of *T. gondii.* Samples collected in winter (January–March), followed by spring and summer, had the highest likelihood to be contaminated than those from autumn. These seasonal differences might be related to the geographical origin of harvested vegetables processed. Precipitation and humidity, excess of surface water run-off and temperatures are variables related with persistence and viability of oocysts that should be recorded for a better interpretation of seasonal variations in oocyst detection [9]. However, despite several studies investigating seroprevalence of antibodies against *T. gondii* in Europe (summarised in [34]), a seasonal variation in incidence of toxoplasmosis was seen only in studies in Austria and Slovenia [35,36]. In both studies, diagnosis of toxoplasmosis cases was highest in the winter, potentially reflecting more infections in autumn.

The results of our study may be used to develop guidelines for testing food matrices for *T. gondii* as recommended by the European Food Safety Agency (EFSA) [5]. Future work should investigate presence of oocysts in different steps of the RTE production to enable implementation of mitigation strategies to reduce or avoid contamination and minimise infection risk for humans; such effort should be focused on the evaluation of the quantity (burden) and viability of the parasites present, the harmonisation of good manufacturing practices (GMP) and the implementation of specific sampling points [37]. Our observation on the potential relevance of RTE salad in *T. gondii* transmission should help improve educational programmes to inform individuals at high risk, such as pregnant people and individuals with a weakened immune system.

## Conclusion

In conclusion, the relative contribution of different environmental matrices as *T. gondii* sources of infection to humans remains largely unknown and the present paper adds information at least concerning *T. gondii* contamination of salad matrices. Our results provide evidence that *T. gondii* oocyst contamination occurs in RTE leafy greens in Europe, which poses a potential risk for consumers. The implemented harmonised analytical procedure could aid in establishing an ISO method and standardise future studies focusing on environmental matrices.

## Data Availability

The data collected and analysed in this study are available in Supplementary Material.

## Supplementary Data

829000 Supplementary Material 1

## Supplementary Data

1027000 Supplementary Material 2

## Supplementary Data

203000 Supplementary Material 3

## Supplementary Data

620544000 Supplementary Material 4
