# Author’s correction for Euro Surveill. 2025;30(22)

**DOI:** 10.2807/1560-7917.ES.2025.30.23.250612c

**Published:** 2025-06-12

**Authors:** Eurosurveillance editorial team

**Affiliations:** 1 European Centre for Disease Prevention and Control (ECDC), Stockholm, Sweden

In the article entitled ‘Molecular detection of Toxoplasma gondii in ready-to-eat salad mixes: multi-country survey using a validated and harmonised standard operating procedure, Europe, 2021 to 2022’ by Calero-Bernal et al., published on 5 June 2025, the affiliations of Rebecca P Berg and Umer Chaudry were incorrect at the time of publication. This mistake was corrected on 11 June 2025 at the request of the authors.

