# Supplementary material for: Molecular detection of Toxoplasma gondii in ready-to-eat salad mixes: multi-country survey using a validated and harmonised standard operating procedure, Europe, 2021 to 2022
Source: Euro Surveill. 2025 Jun 5;30(22):2400594. doi: 10.2807/1560-7917.ES.2025.30.22.2400594 (PMC12143121; doi:10.2807/1560-7917.ES.2025.30.22.2400594)
Supplement: Supplementary Material 1 [file 24-00594_LALLE_Supplement1.pdf]

This supplementary material is hosted by *Eurosurveillance* as supporting information alongside the article "Molecular detection of *Toxoplasma gondii* in ready-to-eat salad mixes in Europe: results from a large multi-country survey using a validated and harmonised standard operating procedure", on behalf of the authors, who remain responsible for the accuracy and appropriateness of the content. The same standards for ethics, copyright, attributions and permissions as for the article apply. Supplements are not edited by *Eurosurveillance* and the journal is not responsible for the maintenance of any links or email addresses provided therein.

This supplementary material contains:

- Supplementary File S1
- Supplementary File S2
- Supplementary Methods
- Supplementary Tables and Figures

Supplementary File S1

STANDARD OPERATING PROCEDURE (SOP) FOR THE IDENTIFICATION OF *TOXOPLASMA GONDII* DNA IN LEAFY VEGETABLE BY PROBE-BASED REAL-TIME PCR

INDEX

1.	**Aim and field of application**	**2**
2.	**Principle of the method**	**2**
3.	**References**	**3**
4.	**Definitions**	**3**
5.	**Devices/instruments**	**5**
6.	**Reagents and chemicals**	**6**
7.	**Procedure**	
7.1	**Sample preparation**	**8**
7.2	**Method**	**8**
8.	**Results**	**17**
9.	**Characteristics of the method**	**18**
10.	**Use of method for “real” samples**	**18**
11.	**Safety measures**	**18**
**Appendix A**		**19**

1. Aim and field of application

This method serves to identify *Toxoplasma gondii* DNA in leafy vegetables by probe-based qualitative real-time PCR (qPCR). This method can be applied to fresh (not frozen) leafy vegetables of a single type or mixed salads that do not contain other ingredients such as non-leafy vegetables.

2. Background and principle of the method

Toxoplasma gondii is a protozoan parasite able to infect all warm blooded animals (mammals or birds), including humans. The sexual stage of the parasite life cycle occurs in felids, the definitive hosts, and the environmentally resistant stage of the parasite, the oocyst, is shed with faeces. Following oocyst ingestion by mammals or birds, the intermediate hosts, the parasite invades different host tissues (especially muscle and brain), where the asexual stage, the tachyzoite, undergoes multiple replication cycles and finally forms tissue cysts containing tens to thousands parasites, named bradyzoites. The cycle is completed when felids eat meat containing *T. gondii* tissue cysts (or oocysts directly). Humans can acquire toxoplasmosis by the ingestion of fruit, vegetables or water contaminated by oocysts or by the consumption of raw or undercooked meat containing tissue cysts (Jones and Dubey, 2012). Usually, toxoplasmosis is asymptomatic, but in immunocompromised persons, it can cause encephalitis, myocarditis, pneumonia, retinochoroiditis and hepatitis. In case of infection acquired during pregnancy, the parasite can be transmitted from the mother to the foetus causing serious foetal diseases, eventually resulting in abortion or to permanent damage that can arise later during adulthood.

Detection of the (oo)cyst stage of protozoan parasites, such as *Toxoplasma gondii* (*T. gondii*), in fresh produce (e.g. leafy vegetables) necessarily requires a first step to elute (oo)cysts from the produce followed by their concentration prior to the detection assay. As reviewed, different procedures have been investigated for each of these stages and for leafy vegetables a paddle-beater approach in glycine buffer is recommended for the elution stage (Lalonde and Gajadhar 2016; Lalle et al., 2018; Lalle et al., 2020) followed by a centrifugation step. Although immunomagnetic separation (IMS) has been reported to concentrate *T. gondii* oocysts using in-house produced antibodies against the oocyst wall (Hohweyer et al., 2016), the procedure cannot be implemented, due to the lack of commercially available and suitable monoclonal antibodies. Moreover, it has been also shown that *T. gondii*-IMS did not improve recovery rate (Hohweyer et al., 2016).

Probe-based real-time PCR is a molecular biology technique that allows the amplification of specific nucleic acid fragments, whose initial and terminal nucleotide sequences are known (oligonucleotide pair). By selecting a specific and unique DNA fragment in the genome of the organism of interest, it is possible to design two oligonucleotides (primer pair), which allow the DNA amplification of the target organism. Furthermore, the technique uses the real-time fluorescence from 5′-3′ exonuclease cleavage of a fluorescently-labelled, target-specific oligonucleotide (hydrolysis probe) to measure DNA amplification at each PCR cycle. The hydrolysis probe incorporates a 5’ reporter fluorophore and a 3’ quencher on a short oligonucleotide complementary to the target sequence. The use of such probes yields increased specificity and sensitivity. Additionally, the assay can be multiplexed allowing the detection of different targets at the same time combining target-specific primer pairs and target-specific probes, each labelled with different fluorophores. Furthermore, a non-target DNA sequence termed an internal amplification control (IAC), can be added in the same sample reaction tube to be co-amplified with the target sequence. Efficiency in IAC amplification allows the presence of PCR inhibitory substances in the sample (derived from the matrix) to be checked, thus helping to distinguish between true and false negatives. When no inhibitors are present, the IAC control signal will always be produced, even when there is no target sequence present. When IAC signal is not produced, the PCR has failed. Probe-based real-time PCR has higher specificity, efficiency and rapidity compared to other

PCR techniques and allows amplification of small traces of target DNA in the matrix.

By real-time PCR, it is possible to detect *T. gondii* DNA from oocysts on vegetables by the simultaneous amplification of specific nucleotide sequences. Specifically two loci, only present in the genome of *T. gondii*, can be targeted: i) the 529 bp repetitive element (529RE) repeated up to 300 times/haploid genome (Homan et al., 2000); ii) and the *B1* gene, present in up to 30 copies/haploid genome (Slany et al., 2019). Further molecular analyses are however necessary to determine the genotype. The IAC, used for this assay, is an artificial DNA sequence consisting of a part of the StTS1 gene (AF483209) from potato (*Solanum tuberosum*) flanked with hybrid primers derived partly from the *Nepenthes* endochitinase gene and partly from the *f57* gene of *Mycobacterium avium* subsp. *paratuberculosis* (MAP), cloned into the pCR 2.1 vector (Slana et al. 2008). This sequence is not even remotely similar to any known organism, and thus, this IAC can be used with any target or sample without the risk of false-positive results.

The food matrix used for the development of this SOP is leafy vegetable mixed salad (containing, in different amounts, green and red baby lettuce, arugula, baby spinach, roman baby lettuce), as this will take into account the different chemical and physical properties associated with various leafy vegetables that could be tested (Lass et al., 2012).

3. References

1. Jones JL, Dubey JP. Foodborne toxoplasmosis. Clin Infect Dis. 2012 Sep;55(6):845-51.
2. ISO/FDI 20837:2006(E). Microbiology of food and animal feeding stuffs – Polymerase chain reaction (PCR) for the detection of food-borne pathogens - Requirements for sample preparation for qualitative detection.
3. ISO/FDI 20838:2006(E). Microbiology of food and animal feeding stuffs – Polymerase chain reaction (PCR) for the detection of food-borne pathogens - Requirements for amplification and detection for qualitative methods.
4. ISO 18744:2016(E). Microbiology of the food chain — Detection and enumeration of *Cryptosporidium* and *Giardia* in fresh leafy green vegetables and berry fruits.
5. Laura F. Lalonde, Alvin A. Gajadhar. Optimization and validation of methods for isolation and realtime PCR identification of protozoan oocysts on leafy green vegetables and berry fruitsFood Waterborne Parasitol. 2016, 2: 1-7.
6. Lalle M, Possenti A, Dubey JP, Pozio E. Loop-Mediated Isothermal Amplification-Lateral Flow Dipstick (LAMP-LFD) to detect *Toxoplasma gondii* oocyst in ready-to-eat salad. Food Microbiol. 2018 Apr;70:137-142.
7. Lass A., Pietkiewicz H., Szostakowska B., Myjak P. The first detection of Toxoplasma gondii DNA in environmental fruits and vegetables samples. Eur J Clin Microbiol Infect Dis 2012, 31:1101–1108
8. Homan WL, Vercammen M, De Braekeleer J, Verschueren H. Identification of a 200- to 300-fold repetitive 529 bp DNA fragment in *Toxoplasma gondii*, and its use for diagnostic and quantitative PCR. Int J Parasitol. 2000 Jan;30(1):69-75.
9. Slany M, Dziedzinska R, Babak V, Kralik P, Moravkova M, Slana I. *Toxoplasma gondii* in vegetables from fields and farm storage facilities in the Czech Republic. FEMS Microbiol Lett. 2019 Jul 1;366(14):fnz170.
10. lana I, Kralik P, Kralova A, Pavlik I. On-farm spread of *Mycobacterium avium* subsp. paratuberculosis in raw milk studied by IS900 and F57 competitive real time quantitative PCR and culture examination. Int J Food Microbiol. 2008 Dec 10;128(2):250-7.

4. Definitions

- **DNA, deoxyribonucleic acid**, polymer of deoxyribonucleotides occurring in a double-stranded (dsDNA) or single-stranded (ssDNA) form.

- **B1**, sequence of the B1 multicopy gene (GenBank AF189871) present at up to 35 copies in the single genome of *T. gondii*.
- **529 Repetitive element (529RE)** is a non-coding nucleotide sequence (GenBank FJ656209) present at up to 300 copies in the single genome of *T. gondii*.
- **IAC**, Internal Amplification Control, DNA added to each reaction in a defined amount or copy number which serves as an internal control for amplification.
- **Oligonucleotide (Primer)**, short sequence of defined length (15-30 nucleotide bases) complementary to a segment of an analytically relevant DNA sequence and used for its amplification.
- **Probe,** fluorescently labelled nucleic acid molecule with a defined sequence used to detect target DNA by hybridization.
- **Hybridization,** specific binding of complementary nucleic acid sequences under suitable reaction conditions.
- **Primer mix**, mix of two or more oligonucleotides of defined length and sequence complementary to a segment of an analytically relevant DNA sequence.
- **Reference DNA,** purified genomic DNA from *T. gondii* (of any genotype) oocysts.
- **Positive process control,** a sample of 30 g ± 1 of leafy vegetable mixed salad experimentally spiked with 100 ± 10 sporulated *T. gondii* oocysts according to the "Spiking guideline from the IMPACT project (in preparation, see Appendix A)". It is analysed in the same working session of test samples, to verify the efficacy of the whole process. <u>NOTE</u> The process includes sample preparation, concentration, DNA extraction and target amplification.
- **Positive control for the DNA extraction,** a leafy vegetable mixed salad pellet spiked with 100 ± 10 sporulated *T. gondii* oocysts. It is analysed at the same time as test samples, to verify the efficacy of the DNA extraction session.
- **Sample DNA,** DNA extracted from a single sample.
- **Positive control for DNA amplification,** a reference DNA; this control is used in the amplification session to verify the efficacy and specificity of the PCR.
- **Negative control for DNA amplification,** reagent grade water; this control is used in the amplification session to verify the efficacy and specificity of the PCR.

The definitions and terminology used in the *EN ISO 22174:2005. Microbiology of food and animal feeding stuffs - Polymerase chain reaction (PCR) for the detection of food-borne pathogens - General requirements and definitions* are applied in the present method.

5. Devices/instruments

- OPTIONAL: Analytical grade water system production, resistivity ≥ 18 Mohm/cm
- Analytical balance, readability 0.1 g
- pH-meter, with ± 0.3 pH accuracy
- Magnetic stirrer with stir bars
- Paddle blender
- Refrigerated centrifuge for 50 mL tubes, up to 2500 x g or more.
- Freezer ≤-15°C
- Bench top bead beater homogenizer for microtubes (e.g. FastPrep 24 Instrument)

- Refrigerated benchtop centrifuge for 1.5-2 mL tubes, minimum 10,000 x g
- Thermoblock (with or without) vibration, temperature range 25-100 °C
- Rotator for 1.5-2 ml microtubes (if using FastPrep kit)
- Refrigerator, temperature range 4±3 °C
- Real-time PCR Instrument with analysis software.
- Adjustable volume pipettes, volume range: 1-10 µL, 2-20 µL, 20-100 µL, 50-200 µL, 200-1000 µL
- Vortex

6. Reagents and chemicals

- **Reagent (analytical) grade water.** Commercially available nuclease-free water, for molecular biology
- **Glycine buffer**. Buffer of 1 M glycine ($C_2H_5NO_2$) pH 5.5. The necessary amount of the buffer is prepared fresh using commercially available glycine powder .

Preparation: Dissolve 75 g of glycine in 800 ml of analytical grade water on a magnetic mixer and adjust the pH to 5.5 ± 0.2 with 1 M HCl or NaOH, then bring to a final volume of 1 L. Store at 4±3°C for maximum 24-48 hours. The glycine powder is stored according to the manufacturer's recommendations.

OPTIONAL: Antifoam agent (e.g. active silicone polymer 100% Antifoam A, Merck A5633) can be added to the glycine buffer at a final concentration of 0.03% if processing leafy vegetables highly rich in saponins, which tend to foam (e.g. spinach). After antifoam addition, stir the buffer vigorously for 30-60 min on a magnetic stirrer before use.

- ***T. gondii* oocysts.** Preparation of purified *T. gondii* sporulated oocysts.
- **Filter paddle-beater bags (holding a volume of 400 ml)**. Various types of bags with filters suitable for volumes up to 400 ml are commercially available. These may have a side filter or an insert central filter bag; either can be used (e.g. VWR 129-0733, VWR 432-3119). NOTE: side filter bags are recommended (e.g. VWR 129-0733).
- **Stand for Paddle-filtered bags**. Stands are commercially available. NOTE. A plastic box of suitable size or similar can also be used.
- **DNA extraction kit**. Commercial kit allowing sample processing with a bead-beating step (e.g., FastDNA Spin kit for Soil, MP Biochemicals, or DNeasy PowerSoil Kit / DNeasy PowerSoil Pro kit, Qiagen). If no bead beating instrument is available, then a commercial kit based on thermal treatment of the sample with proteinase K and DNA purification by silica columns can be used (e.g. QIAamp Fast DNA Stool Mini, Qiagen). However, sensitivity may be lower and this should be established.
- **DNA fish sperm solution**. Commercially available. (e.g. catalogue 18580.01, Serva Electrophoresis GmbH), at final concentration of 62.5 g/l.
- **Centrifuge tubes.** Commercially available molecular biology grade tubes (0.2 mL, 1.5 mL, 2 ml) and 50 mL conical tubes.
- **Polyethylene backed absorbent paper**. Commercially available and used to soak up spills and prevent flow through to the work surface.
- **Hot-start PCR Probe Master Mix**. Commercial reagent designed for use with probe-detection technology, employing a hot-start DNA polymerase and compatible with real-time multiplex PCR format assay (e.g. SensiFAST™ Probe Kit, Bioline). NOTE: depending on the real-time PCR instrument in use, a master mix with the passive inert fluorescent dye carboxyrhodamine (ROX) must be used for fluorescence signal normalization (e.g. SensiFast™ Probe Lo-ROX Kit, Bioline)

- **Real-time PCR consumables.** Commercially available disposable vials or plates compatible with the real-time PCR instrument.
- **Primers (Oligonucleotides).** Commercial preparation (*Table A*); the lyophilized product is reconstituted with reagent grade H_2O, at the appropriate concentration (*Table A*).
- **IAC:** an artificial DNA sequence consisting of a part of the StTS1 gene (AF483209) from potato (*Solanum tuberosum*) flanked with hybrid primers derived partly from the *Nepenthes* endochitinase gene and partly from the *f57* gene of *Mycobacterium avium* subsp. *paratuberculosis* (MAP), cloned into the pCR 2.1 vector (Slana et al. 2008). The plasmid pDrive (Qiagen) (3.85 kb vector and 141 bp insert, with ampicillin and kanamycin resistance) is available upon request to VRI. IAC stock solution ($1x10^{10}$ copies/µl) can be stored at -20°C in Tris-EDTA pH 8.0 (TE) buffer for up to 10 years. Working solution ($5x10^4$ copies/µl) can be stored in TE buffer at -20°C for up to 5 years. It is recomended to prepare small aliquots of the IAC to avoid frequent freeze/thaw cycles.
- **Hydrolysis probes:** Commercial preparation (*Table A*); the lyophilized product is reconstituted with reagent grade H_2O at a concentration of 100 pmol/µL according to the manufacturer's recommendations; the lyophilized product can be stored frozen for up to 20 years; the reconstituted product can be stored frozen up to 10 years.
- **Positive process control,** a sample of 30 g ± 1 g of mixed leafy green vegetable experimentally spiked with 100 ± 10 *T. gondii* sporulated oocysts according to the "Spiking guideline from the IMPACT project (Annex A)". It is analysed at the same time as test samples, to verify the efficacy of the whole process.
- **Positive control for DNA extraction:** leafy vegetable mixed salad pellet spiked with 100 ± 10 *T. gondii* sporulated oocysts. It is analysed at the same time as test samples, to verify the efficacy of the DNA extraction session. Store at -20 °C for up to 5 years.
- **Reference DNA/Positive control for DNA amplification.** Purified genomic DNA from *T.gondii* sporulated oocysts (for calculation consider that 70 fg of DNA/sporozoite = 560 fg of DNA/oocyst); store at -20 °C for up to 5 years.
-

Table A. Primer and probe sequences.

**Primers and Probes**	**Sequence 5'-3'**[b]
529RE Forward primer	5′-AGGAGAGATATCAGGACTGTAG-3'
529RE Reverse primer	5′-GCGTCGTCTCGTCTAGATCG-3'
529RE HEX Probe	5´-HEX-CCGGCTTGGCTGCTTTTCCT-MGBEQ-3´
B1 Forward primer	5′-TCGAAGCTGAGATGCTCAAAGTC-3′
B1 Reverse primer	5′-AATCCACGTCTGGGAAGAACTC-3′
B1 FAM primer	5′-FAM-ACCGCGAGATGCACCCGCA-MGBEQ-3′
IAC[a] Forw primer	5'-AGAGGACCGGGATATTCGAC-3'
IAC Rev primer	5'-AGGTAGTCCGAGGAAAACTCTAAAC-3'
IAC Cy5 probe	5'-Cy5-AGGCTCTTCTATGTTCTGACCTTGTTGGA-MGBEQ-3'

[a] Probe quenchers can be also BHQ.

[b] The IAC is plasmid DNA (pDNA) as described in (Slany et al., 2019). Available upon request.

7. Procedure

7.1 Sample preparation

The sample (30 g ± 1g in sealed plastic bags) should be inspected upon receipt to check the integrity of the packaging to ensure that there has been no likelihood of cross-contamination between samples. In addition, the condition of the sample (freshness, leaf integrity etc.) should be noted. If the sample is in poor condition or there is the possibility of cross-contamination between samples then this should be recorded and reported that the efficiency of the test may be compromised. Please refer to further comments in section 10 at the end of this document.

Samples are then stored refrigerated at 4°C for a maximum of 3 days before being tested.

7.2 Method

7.2.1 ***Recovery of T. gondii oocysts from test sample***

- If not otherwise specified, the procedure is carried out at room temperature.
- Before starting the procedure, prepare a sufficient volume of glycine buffer for the number of samples to be analysed.

a) Weigh the sample into the filter bag and label as appropriate.

b) Hold the bag upright, either by placing it on a stand or using another support for the bag, such as a plastic box.

c) Add 200 ml glycine buffer.

d) Fold the upper edge of the bag.

e) Accommodate the bag in the paddle homogenizer.

f) Process sample for 30 s at 300 rpm. If possible, record the beater speed (speeds of 200-300 rpm have been found effective). NOTE: Depending on the paddle homogenizer and type and condition of the sample, optimal paddling conditions (time and speed) must be established to avoid over-smashing of the leaves that would eventually result in large vegetable pellets at the end of the recovery process (see step "x") not suitable for DNA extraction. For baby leaves, 15 s at 300 rpm have been found to be effective.

g) Check leaves are still immersed in liquid (shake down) and repeat step (f).

h) Carefully, transfer the bag from the paddle to the stand.

i) Prepare four 50 ml conical tubes for each test sample and mark them with the number corresponding to the test sample.

j) Collect homogenate by: i) pouring into the 50 ml conical tubes, ensuring that all the vegetable matter is retained in the filter; or ii) pouring into a beaker (250 mL volume) and then divide the homogenate in 4 x 50 ml conical tubes (NOT recommended); or iii) by pipetting from bag into 50 ml conical tubes (this can prevent risk of solution spillage). Gently squeeze/manipulate the bag and the filter in order to ensure that all the eluate is obtained from the sample. Equally distribute the homogenate solution into the four 50 ml conical tubes. NOTE: The following steps from k) to x) describe concentration of the eluate to an appropriate volume for DNA extraction. With the exception of centrifugation time and speed, you can adapt according to the size of the resulting vegetable pellet.

k) Centrifuge the tubes at 2500 x g for 10 min at 4°C.

l) After centrifugation, remove the supernatant from the 50 ml tubes leaving 1-2 ml of solution and ensuring the pellet is not disturbed. If no pellet is visible, extra care shall be taken to ensure that parasites are not lost during aspiration.

m) Resuspend the pellet in the residual liquid left in the bottom of each tube by gentle vortexing or shaking by hand and combine the pellets into a single centrifuge tube. Rinse the tubes with a further 10 ml of Glycine buffer and pool everything together with the vegetable pellet.

n) Add 10 ml of glycine buffer to the paddle filter bag and rinse by manipulating the sample from outside the bag. Add the rinsate from the sample to the 50 ml tube of step "m".

o) Repeat step "n".

p) Centrifuge the tubes at 2500 x g for 10 min at 4°C.

q) After centrifugation, remove the supernatant from the 50 ml tubes leaving 1-2 ml of solution and ensuring that the pellet is not disturbed. If no pellet is visible, extra care shall be taken to ensure that oocysts are not lost during aspiration.

r) Add 50 ml of reagent grade water or Milli-Q water to wash the pellet.

s) Centrifuge the tubes at 2500 x g for 10 min.

t) After centrifugation, remove the supernatant from the 50 ml tubes leaving 1 ml of solution and ensuring the pellet is not disturbed. If no pellet is visible, extra care shall be taken to ensure that parasites are not lost during aspiration.

u) Resuspend the pellet in the residual volume by pipetting up and down and transfer into a new 2 ml tube correctly numbered. Rinse the 50 ml tube with 1 ml of reagent grade water or Milli-Q water and combine in the 2 ml tube,

v) Centrifuge the tubes at 2500 x g for 10 min at 4°C.

w) After centrifugation, remove all the supernatant from the 2 ml tubes ensuring the vegetable pellet is not disturbed. If no pellet is visible, extra care shall be taken to ensure that parasites are not lost during aspiration. NOTE. To ensure DNA extraction can occur in a single tube, vegetable pellet size should not exceed 0.5 mL. If it is larger than 0.5 mL, then divide into 2 or more (as appropriate) lots. If the pellet is excessively large due to particular characteristics of the sample, please refer to section 10 at the end of the document.

x) If necessary, the pellet can be stored at -20°C before proceeding with DNA extraction. NOTE. If DNA extraction is performed with FastDNA™ Spin Kit for Soil (see below) prior to freezing, the pellet can be resuspended in resuspension buffer (Sodium Phosphate Buffer, FastDNA Spin kit for Soil, MP Biochemical) to reach a final volume of approximately 900 µL. For DNeasy PowerSoil kit (Qiagen) or DNeasy PowerSoil Pro Kit (Qiagen) users, transfer and perform the last centrifugation step (step v) in a 2 ml screw cap tube compatible with the bead beating homogenizer and then freeze the sample.

7.2.2 ***DNA extraction***

- If not otherwise specified, the procedure is carried out at room temperature.
- For DNA extraction from a frozen pellet, ensure that the sample is completely thawed.
- Each working session requires the DNA extraction of a "reference sample" identified as "positive control for the extraction".

Two protocols for DNA extraction based on bead-beating are provided below. NOTE: Other comparable DNA extraction kits can be used but were not tested when this SOP was developed.

If no bead-beating instrument is available, a third DNA extraction protocol including a pre-treatment of the sample with several freeze/thaw cycles is provided. NOTE. It has been experimentally verified that this last protocol without bead-beating might have a lower efficiency in extracting *T. gondii* DNA, and this is expected to reduce the sensitivity of the overall detection method.

7.2.2.1 DNA extraction with FastDNA™ Spin Kit for Soil (MP Biochemicals, catalogue number SKU 116560200-CF)

a) Thaw the tubes containing the sample at room temperature. NOTE. Proceed directly to step (c) if pellet has already been resuspended in the resuspension buffer prior to freezing.

b) Add a sufficient amount of resuspension buffer (Sodium Phosphate buffer, FastDNA Spin kit for Soil, MP Biochemical) to reach a final volume of approximately 900 µL.

c) Add 125 µL of homogenization buffer (MT buffer, FastDNA Spin kit for Soil, MP Biochemicals) to an appropriately numbered lysis tube (Lysis Matrix E tube, FastDNA Spin kit for Soil, MP Biochemicals)

d) Transfer the sample from step "b" to the lysis tube.

e) Homogenise the sample at an amplitude of 6.0 for 40 sec. on a benchtop bead beater instrument (FastPrep Instrument). NOTE: if a different bead beater instrument is used, an appropriate setting should be experimentally established.

f) Repeat step "e".

g) Centrifuge the tube at 14,000 x g for 10 minutes at 4°C to pellet debris.

h) In the meantime, add 250 µL of protein precipitation buffer (buffer PPS, FastDNA Spin kit for Soil, MP Biochemicals) to a clean appropriately numbered 2.0 ml microcentrifuge tube.

i) Transfer the supernatant from step "g" to the 2.0 ml microcentrifuge tube containing the PPS buffer and mix by inverting the tube for 10 times.

j) Centrifuge the tube at 14,000 x g for 5 minutes.

k) In the meantime, resuspend the silica matrix (Binding Matrix, FastDNA Spin kit for Soil, MP Biochemicals) by vortexing for 30 sec and add 1 ml of resuspended silica matrix to a clean 2.0 ml microcentrifuge tube properly numbered.

l) Transfer the supernatant from step "j" to the 2 ml microcentrifuge containing the silica matrix and mix gently on a rotary shaker for 2 min.

m) Place the microcentrifuge tube in a rack for 3 min to allow setting of the silica matrix.

n) Carefully remove and discard 500 µl of supernatant without disturbing the silica matrix.

o) Resuspend by pipetting the silica matrix in the remaining supernatant.

p) Transfer 500 µl of the mixture to a properly numbered collection column positioned on a collecting tube.

q) Centrifuge at 14,000 x g for 1 minute.

r) Empty the collecting column and repeat steps from "p" to "r" until all the matrix is transferred to the collection column.

s) Add 500 µl of washing buffer (SEWS-N, FastDNA Spin kit for Soil, MP Biochemicals) to the collection column and gently resuspend matrix by pipetting.

t) Centrifuge the collection column at 14.000 x g for 1 minute.

u) Empty the collecting tube.

v) Centrifuge the collection column at 14,000 x g for 2 minutes to dry the matrix.

w) Transfer the collection column to a new appropriately numbered collecting tube .

x) Air dry the collection column for 5 minutes.

y) Gently resuspend the silica matrix with 100 µl of elution buffer (DES FastDNA Spin kit for Soil, MP Biochemical) by pipetting. NOTE. DES=molecular grade H_2O

z) Incubate at 55°C (±3°C) for 5 minutes on a thermoblock.

aa) Elute DNA by centrifugation at 14,000 x g for 1 minute, discard the collection column and keep the collecting tube

with DNA

bb) The obtained DNA will be defined as 'DNA/sample' and can be stored frozen (≤-20°C) for up to 5 years.

7.2.2.2 DNA extraction with DNeasy PowerSoil Kit (Qiagen, Catalogue number 12888-50)

Important points before starting:

- All steps can be performed at room temperature, if not mentioned otherwise
- If Solution C1 is precipitated, heat solution to 60°C until the precipitate has dissolved before use.
- Prepare a thermoblock at 70°C for use in step "r"

NOTE: The kit has now been replaced with "DNeasy PowerSoil Pro Kit (Qiagen, Catalogue number 47014 or 47016).

a) Thaw the pellet at RT.

b) Add to 1.2 ml of bead solution to the sample.

c) Add 60 µl of solution C1 to the sample and transfer to PowerBead Tubes (provided) and gently vortex to mix (5 s).

d) Add 6.25 µg (10 µl solution of fish sperm DNA and mix quickly)

e) Homogenise in MagnaLyser (or other suitable bead beater) at 6400 rpm for 60 s. NOTE: if a different bead beater instrument is used, an appropriate setting should be experimentally established.

f) Centrifuge tubes at 10,000 x g for 1 min at room temperature.

g) Transfer the supernatant (500 µl maximum) to a clean 2 ml Collection Tube (provided)

h) Add 250 µl of Solution C2 and vortex for 5 seconds. Incubate on water and ice at 4°C for 5 minutes.

i) Centrifuge the tubes at room temperature for 1 minute at 10,000 x g.

j) Transfer up to 750 µl of supernatant to a clean 2 ml Collection Tube (provided). Avoid transferring the pellet (full of organic and inorganic matter). NOTE: supernatant could be eventually transferred to a 5 ml or similar vial/tube (not provided in the kit) instead of a 2 ml Collection Tube before proceeding with step "k".

k) Add 1200 µl of Solution C4 to the supernatant and vortex for 5 seconds. NOTE: extra care must be taken due to the high volume (1950 µl full and spillover can easily happen).

l) Centrifuge briefly (short spin; 5 s) to remove drops from the inside of the tube lid.

m) Load approximately 675 µl onto a Spin Filter and centrifuge at 10,000 x g for 1 minute at room temperature. Discard the flow through.

n) Two-times repeat step "m" (A total of three loads for each processed sample are required).

o) Discard the flow through and centrifuge at 10,000 x g for 1 minute at room temperature.

p) Add 500 µl of Solution C5 and centrifuge at room temperature for 30 seconds at 10,000 x g.

q) Transfer the Spin Filter into a clean 2 ml Collection Tube (provided). Avoid splashing any Solution C5 onto the Spin Filter.

r) Add 100 µl of preheated (70°C) Solution C6 (or TE buffer) to the centre of the white filter membrane. Incubate for 3 min at laboratory temperature, then centrifuge at 10,000 g for 1 min to elute DNA.

s) Reload the filtrate to the membrane, incubate 1 min and centrifuge at 10,000 g for 1 min to elute DNA.

t) The obtained DNA will be defined 'DNA/sample' and can be stored frozen (≤-20°C) for up to 5 years.

7.2.2.3 DNA extraction with QIAamp Fast DNA Stool Mini Kit (Qiagen, catalogue number 51604)

IMPORTANT: If bead-beater equipment is not available, the following protocol could be used. However the efficiency of DNA extraction from *T. gondii* oocysts using this protocol is lower.

Important points before starting:

- All centrifugation steps should be carried out at room temperature (15–25°C) at 20,000 x g (approximately 14,000 rpm). Increase the centrifugation time proportionately if your centrifuge cannot reach 20,000 x g (e.g., instead of centrifuging for 5 min at 20,000 x g, centrifuge for 10 min at 10,000 x g).
- Prepare a thermomixer/heating block with adapters to accommodate 2 ml vials or a water bath at 95°C.
- Redissolve any precipitates in Buffer AL and InhibitEX Buffer by incubating at 37–70°C.
- Add ethanol to Buffer AW1 and Buffer AW2 concentrates.
- Mix all buffers before use.

a) Thaw the sample and perform 5 cycles each of 5 min in liquid N_2 followed by 5 min at 95°C.

b) Add 1 ml InhibitEX Buffer to each sample. Vortex continuously for 1 min or until the sample is thoroughly homogenised. NOTE: It is important to vortex the samples thoroughly. This helps ensure maximum DNA concentration in the final eluate.

c) Heat the suspension in the thermomixer with shaking for 10 min at 95°C. Vortex for 15 s. After this step, the rest of the procedure can be performed at room temperature (15–25°C).

d) Centrifuge the sample at full speed for 1 min to pellet vegetable particles. IMPORTANT: Do not transfer any solid material. If particles are still visible in the supernatant, centrifuge the sample again.

e) Pipette 45 µl proteinase K (provided in the kit) into a new 2 ml microcentrifuge tube (not provided).

f) Pipette all the supernatant from step “d” into the 2 ml microcentrifuge tube containing proteinase K.

g) Add 1 mL Buffer AL and vortex for 15 s. Note: Do not add proteinase K directly to Buffer AL. It is essential that the sample and Buffer AL are thoroughly mixed to form a homogeneous solution.

h) Incubate in the thermomixer with shaking at 70°C for 10 min. Centrifuge briefly (short spin; 5 s) to remove drops from the inside of the tube lid (optional).

i) In the meantime, add 1 mL of ethanol (96–100%) into a new appropriately numbered 5 ml (or bigger) vial.

j) Add supernatant from step “h“ to the 5 ml vial containing ethanol and mix by vortexing. Centrifuge briefly to remove drops from the inside of the tube lid (optional).

k) Carefully apply 600 µl lysate from step “j” to the QIAamp spin column. Close the cap and centrifuge at full speed for 1 min. Place the QIAamp spin column in a new 2 ml collection tube, and discard the tube containing the filtrate. Close each spin column in order to avoid aerosol formation during centrifugation. If the lysate has not completely passed through the column after centrifugation, centrifuge again until the QIAamp spin column is empty.

l) Repeat step j) until all the lysate has been used (5x).

m) Carefully open the QIAamp spin column and add 500 µl Buffer AW1. Centrifuge at full speed for 1 min. Place the QIAamp spin column in a new 2 ml collection tube, and discard the collection tube containing the filtrate.

n) Carefully open the QIAamp spin column and add 500 µl Buffer AW2. Centrifuge at full speed for 3 min. Discard the collection tube containing the filtrate. Note: Residual Buffer AW2 in the eluate may cause problems in downstream

applications. Some centrifuge rotors may vibrate upon deceleration, causing the flow-through containing Buffer AW2 to come in contact with the QIAamp spin column. Removing the QIAamp spin column and collection tube from the rotor may also cause flow-through to come into contact with the QIAamp spin column.

o) Place the QIAamp spin column in a new 2 ml collection tube (not provided) and discard the old collection tube with the filtrate. Centrifuge at full speed for 3 min. This step helps to eliminate the chance of possible Buffer AW2 carryover.

p) Transfer the QIAamp spin column into a new, labelled 1.5 ml microcentrifuge tube (not provided) and pipette 100 µl of molecular grade H_2O (or Buffer ATE) directly onto the QIAamp membrane. Incubate for 1 min at room temperature, then centrifuge at 10,000 g for 1 min to elute DNA. OPTIONAL Reload the eluate to the membrane, incubate for 1 min and centrifuge at 10,000 g for 1 min to elute DNA.

q) The obtained DNA will be defined as 'DNA/sample' and can be stored frozen (≤-20°C) for up to 5 years.

7.2.3 ***Real-time PCR amplification***

- Unless otherwise clearly stated, store tubes on ice; use filter tips and wear disposable gloves.
- "In each real-time PCT run, use a positive and a negative amplification control. Use reference DNA as a positive control and water as a negative control.
- For quantification use a 10-fold serial dilution of quantified reference DNA.
- All DNA samples shall be run in triplicate, including positive and negative controls.

a) Thaw DNA/sample, IAC, PCR master mix, primers, probes and positive amplification controls (Reference DNA).

b) Label tubes or wells as appropriate.

c) Prepare an adequate volume of the amplification mix for all samples and controls. Determine the volume on the basis of a single sample amplification mix (*Table B*) and the total number of samples plus 3 (1 for the positive amplification control, 1 for the negative one, 1 for an extra reaction).

Table B. Single sample amplification mix: components and volumes

**Component**	**Volume (µl)**
PCR master mix	12.5 µL
B1 Forward primer [50 µM]	0.125 µL
B1 Reverse primer [50 µM]	0.125 µL
B1 5-FAM Probe [20 µM]	0.125 µL
529RE Forward primer [50 µM]	0.125 µL
529RE Reverse primer [50 µM]	0.125 µL
529RE 5-HEX Probe [20 µM]	0.185 µL
IAC Forward primer [50 µM]	0.125 µL
IAC Reverse primer [50 µM]	0.125 µL
IAC CY5 probe [20 µM]	0.185 µL
IAC [$5x10^4$ copies/µl]	0.06 µL
Nuclease free H_2O	up to volume
Template DNA	2-10[a]
Total volume:	25

[a] For the DNA/sample use 10 µl of an undiluted DNA preparation. For reference DNA use 2 µL as template volume.

d) Mix the amplification mix by vortexing and, if necessary, centrifuge at maximum speed for a few sec.

e) Transfer the appropriate amount (µL) of the amplification mix into each tube/well (step “b”).

f) Add up to 10 µL of the DNA/sample to be tested to each tube. For reference DNA use 2 µL and adjust the volume with reagent grade water.

g) Close the tubes or seal the plate with sealing foil and centrifuge at maximum speed for a few sec to ensure the mix is at the bottom of the tube/well.

h) Set the cycling conditions on the real-time PCR instrument (*Table C*) and start the amplification.

Table C. Reaction conditions

Stage		Temperature	Duration (sec)	Ramping rate[a]	Comments
Activation		95 °C	According to Master Mix polymerase	According to equipment	
Cycling (45 cycles)	Denaturation	95 °C	15	4.4	
	Annealing/ Extension	60 °C	40	2.2	Acquiring fluorescence on appropriate channels

[a] *Not all real-time PCR equipment allow setting of this parameter or may require a different setting to defined during SOP implementation*

7.2.4 <u>*Result display and interpretation*</u>

Comments on data analysis and validity of the real-time PCR run are given in the following table:

**Analyte**	**Comments**
Fluorophore	Analyse data from the HEX (light green λ_{ab} 535- λ_{em} 556), FAM (dark green, λ_{ab} 495- λ_{em} 516) and Cy5 (red λ_{ab} 650- λ_{em} 670)
IAC	Amplification signal is present in all negative and positive samples with comparable Ct values (±1 Ct value). <u>NOTE:</u> In samples highly positive for *T. gondii* DNA, the Ct value for the IAC could be higher compared to the negative controls.
Slope correct (if applicable)	Apply the (noise) slope correction to normalise against background fluorescence.
Apply threshold	Set the fluorescence threshold so that it crosses the area where the amplification plots (logarithmic view) are parallel (the exponential phase) and the precision of the replicates is highest. Set the threshold just above the background amplification signal.
Ct values	No amplification signals should be visible in negative controls = NTC. Ct values of positive controls should lie in an appropriate range. Apply personal upper limits if required.
Amplification curve	All curves should be sigmoidal and reach raw fluorescence in a range acceptable according to instrument characteristics.
Reaction dynamics	R^2= greater than 0.94 (linearity), E= 0.85-1.10 (efficiency)
Targets	Amplification signal for both targets is absent in negative controls and it is below a Ct value of 39 in positive control. NOTE: lower Ct cut-off values for each target gene might be obtained due to DNA polymerase and equipment performance.

8. Results

If the test is considered valid, the results are expressed in the test report as described below (see Table D for quick interpretation). Always report the obtained Ct values and the threshold settings.

- POSITIVE for the presence of *T. gondii* DNA, if the amplification signal of at least one target (B1 or 529RE) for one or more sample replicates produced is below a Ct value of 39 AND the IAC reaction is positive. <u>NOTE</u>: In samples highly positive for *T. gondii* DNA, the Ct value for the IAC could be much higher, or even negative, compared to the positive and negative controls.
- NEGATIVE if the amplification signal for both targets (B1 and 529RE) in all sample replicates is absent or above a Ct value of 39 AND Ct values for the IAC are comparable (±1-2 Ct value) with the positive and negative controls.
- NOT DETERMINED (e.g. inhibited reaction) if amplification signal for both targets (B1 and 529RE) in all sample replicates is absent or above a Ct value of 39 and Ct values for IAC are above 39 or generally much higher (Ct>3) compared to the positive and negative controls. <u>NOTE</u>: In this event, it might be useful to either use a lower amount of input DNA (i.e. 5 µl) or to dilute the DNA sample (e.g. 1:2-1:5) and repeat the test. During SOP development and validation, amplification of vegetable samples spiked with 10 oocysts could be achieved even using 2 µl of extracted DNA.

Table D. Interpretation of real-time PCR results

**B1 Target (FAM chanel)**	**529 RE Target (HEX chanel)**	**IAC (Cy5 chanel)**	**Result interpretation**
positive	positive	Positive/negative	positive for *T. gondii*
positive	negative	Positive/negative	positive for *T. gondii*
negative	positive	Positive/negative	positive for *T. gondii*
negative	negative	positive	negative for *T. gondii*
negative	negative	negative[a]	not determined

[a] Or if Ct values is much higher (Ct>3) compared to the positive and negative controls

9. Characteristics of the method

This method has been characterised in terms of repeatability and specificity with a Limit of detection of DNA equivalent to 10 *T. gondii* oocysts/30 g of leafy vegetables mixed salad (LoD of 0.01 oocyst DNA equivalent/reaction) with a 100% sensitivity (N=12) using the bead-beating-based DNA extraction protocol.

10. Use of method for “real” samples

This method can also be applied to samples provided for investigation that may not be in ideal condition (e.g. deteriorated due to prolonged storage or non-refrigerated storage, treated or exposed to other conditions such as salad dressing, etc.). In this case, sample quality should be recorded upon receipt as well as any necessary deviation/adjustment of this SOP. For example, for some samples the pellet produced by paddle beating as described may be too large to include as either a single or two sub-samples for DNA extraction. In this case, either a proportion of the sample could be used or further samples could be subjected to shorter paddle beating cycles. Such modifications must be clearly stated in the report and it should be clarified that the limit of detection may be higher than stated here.

11. Safety measures

This method must be carried out only by authorised and trained personnel. The operator should wear individual protection devices during the test performance. For general safety measures, refer to the CDC guidelines

Appendix A

Procedure for artificial oocyst contamination of salads

1. Prepare sufficient working suspension (10^4-10^5/ml) of purified *T. gondii* oocysts by diluting an appropriate volume of stock suspension in sterile phosphate buffer saline (PBS) to obtain the concentrations required for the experiments.
2. Enumerate the working suspension by microscopy performing a number of counts (at least in triplicate) on an aliquot of suspension (typically 10-20 µl) in hemocytometer.
3. Prepare sufficient spiking suspension (e.g. 10^3/ml for 100 oocyst in 100 µl) and count it 10 times by spotting onto a microscope slide (single well, 9 mm, or triple well, 3 mm).
4. Air dry.
5. Add 10 µl of methanol per well, and air dry.
6. Count the number of oocysts under a microscope using visible and UV light. NOTE. Oocysts can sometimes be found sometimes outside the edge of the well!
7. Calculate the mean number of oocysts per slide, the standard deviation and the coefficient of variation (CV).
8. Record all counts. An acceptable coefficient of variation (CV) is ideally < 10 % for bulk oocysts, and ≤ 20 % for inocula with small numbers of oocysts.
9. Store at 5 ± 3 °C and use the final suspension to inoculate the matrix within 24 h of preparation. Vortex the oocyst suspension for 30 s and then dispense 100 µl into each filter bag containing the matrix samples, as follow:
10. Place and weigh 30 g ± 1g of leafy vegetable sample in a smooth filter bag, using tweezers if necessary.
11. For an artificially uncontaminated negative control sample dispense 100 µl oocyst-free buffer in 10 droplets of 10 µl each distributed across different areas of the sample
12. For spiked samples, dispense 100 of oocyst suspension in 10 droplets of 10 µl each distributed across different areas of the sample.
13. Aim for the centre of leaves to ensure that the spike suspension does not run off
14. Leave to dry at room temperature for 2-3 h with the bag open
15. Close the bag and place at 4°C overnight
16. Process as per selected protocol.
17. To verify the amount spiked, for every third sample inoculated, as a minimum, dispense 100 µl onto a microscope slide (single well, 9 mm, or triple well, 3 mm).
18. Air dry.
19. Add 10 µl of methanol per well, and air dry.
20. Count the number of oocysts under a microscope using sible and UV light. NOTE. Oocysts can sometimes be found outside the edge of the well!
21. Calculate the mean number of oocysts per slide, the standard deviation and the coefficient of variation (CV).

Supplementary File S2

A PAN-EUROPEAN RING TRIAL TO VALIDATE THE TOXOSOURCES STANDARD OPERATING PROCEDURE FOR THE "MOLECULAR DETECTION OF T. GONDII OOCYSTS CONTAMINATION IN READY-TO-EAT SALADS BY MULTIPLEX QPCR"

1. ***Purpose***

In the course of Year 3 of the TOXOSOUCES Joint Research Project, the Standard Operating Procedure (SOP) "Molecular detection of *T. gondii* oocysts contamination in ready-to-eat salads by multiplex qPCR", was prepared. This was implemented in the first half of Year 4 in TOXOSOURCES consortium partner laboratories that would be involved in the multicentre "pilot survey". The SOP has been developed to assess the presence of *T. gondii* oocyst contamination in leafy green vegetables (mixed salad) by detecting *T. gondii* genomic DNA using a hydrolysis probe-based qualitative multiplex real-time PCR targeting both the B1 gene and the 529 repetitive element. The aim of this ring trial (RT) was to evaluate the performance of laboratories in detecting the presence of *T. gondii* oocysts in leafy green vegetables (mixed salad) and to provide a solid base for the delivery of homogeneous and reliable data in the course of the "pilot survey". Data from the RT will also provide further validation of the SOP taking into account the different steps of the procedure (oocyst recovery from the matrix, DNA extraction and qPCR).

2. ***Items description***

RT items consist of three panels of samples:

✔ Panel 1: 5 vials containing (or not) enumerated *T. gondii* oocysts suspension in sterile H_2O (supplemented with antibiotic/antimycotic) to be used for spiking of leafy salads. **Each laboratory should individually perform spiking of the vegetable matrix using samples of Panel 1 as detailed below;**

✔ Panel 2: 5 vials containing vegetable sediment spiked with *T. gondii* oocyst suspension or buffer without oocysts;

✔ Panel 3: 3 vials containing gDNA of vegetable sediment spiked with or *T. gondii or buffer without oocysts*.

All vials are individually labeled with distinct numeric codes to ensure anonymity of samples. Samples will be provided refrigerated!

IMPORTANT!

Upon arrival in the lab, the packaging and its contents must be checked for correctness and completeness. The samples should be immediately stored as follows:

- **Panel 1, at +4°C until the spiking will be done (whenever possible within 24-48h);**
- **Panel 2 and panel 3, at -20°C until the test is performed (DNA extraction and qPCR).**

3. **Additional material provided:**

- **Positive control DNA** (2.8 ng/µl=5000 oocyst/µl gDNA from in vitro cultured *Toxoplasma gondii* RH tachyzoites) to prepare the standard curve (see the Table below).
- **IAC** ($5x10^8$ copies/µl in TE). For testing in qPCR it must be diluted to $5x10^4$ copies/µl in TE!

Both control DNA and IAC should be enough for the experimental survey!

Table 1. How to prepare gDNA serial dilution for the standard curve

**Dilution series Tachyzoites gDNA**	**Tachyzoites gDNA conc/µl**	**Theoretical oocysts/µl**		**Input DNA 2µl**
T1	2.8 ng/µl	$0.5x10^4$	5000	10,000
T2	280 pg/µl	$0.5x10^3$	500	1000
T4	28 pg/µl	$0.5x10^2$	50	100
T4	2.8 pg/µl	0.5x10	5	10
T5	280 fg/µl	0.5	0.5	1
T6	28 fg/µl	0.05	0.05	0.1
T7	2.8 fg/µl	0.005	0.005	0.01

4. ***Material to be provided by RT participant***

In addition to what is stated in "RT items description" no other material, reagents or consumables will be provided by the RT organizer.

As a matrix for spiking, use a commercially **ready-to-eat green baby lettuce (monotype and NOT organic product!)**. Use at least 200 g of the same brand and batch (lot) with an appropriate best-by date (to ensure the analysis is done before expiration date). If the salad bag size is smaller than 200 g, ensure to mix the different bags together to obtain a homogeneous matrix.

NOTE: These products are available in France, Spain, Germany, Italy, Poland, Czech Republic, Denmark and UK.

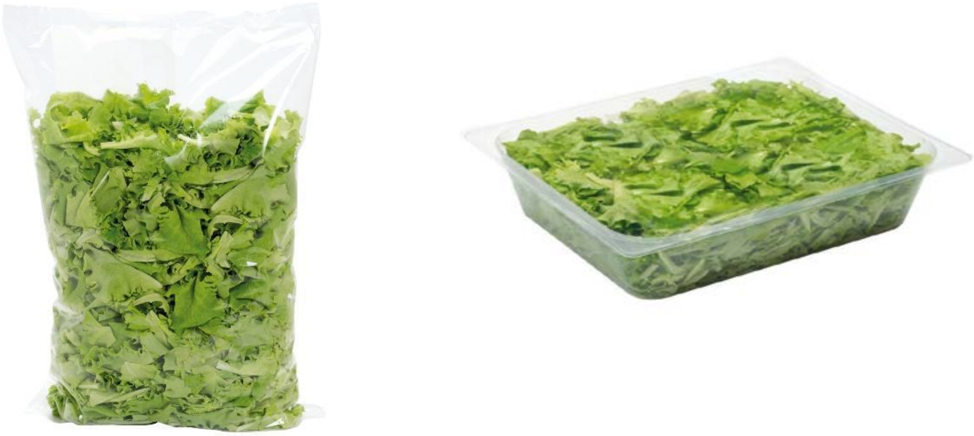

Figure 1. Examples of RTE green baby lettuce.

For preparation of the qPCR standard curve the RT participant should use ten times serial dilutions of their own *Toxoplasma gondii* genomic DNA according to the SOP.

5. ***RT Procedure***

The RT participants will be informed in advance by email about the date of delivery of the RT items. The items will be sent on a Monday to ensure the package is delivered to the participant within the week.

On the day of shipping, the participant will receive an individual laboratory code and a link to an on-line form were the following information must be reported:

1. Participant information and RT item checklist
2. Materials and Methods used to analyze RT samples
3. Results

The on-line form will remain active up to the RT deadline (deadline will be indicated in the email to the participant), after this date results will no longer be accepted.

In addition, a protected Excel file will be provided to report in detail the RT results (e.g., Ct value, qPCR reaction dynamic parameters).

The analysis should be done in compliance with the SOP. Use of reagents other than those detailed in the SOP (e.g. DNA extraction kit; qPCR Mastermix) is acceptable providing that their characteristics are consistent with the expected performance of the test.

The following should be taken into account:

i) For DNA purification, it is recommended to use kits based on a bead-beating step that are more efficient compared to other systems. If a different extraction method (e.g. kits based on silica-membrane) is used, include several freeze and thaw cycles before DNA extraction as indicated in the SOP (paragraph 7.2.2.3);

ii) For **the qPCR, all DNA samples shall be run in triplicate**, including positive and negative controls and the standard curve.

iii) **NOTE: for the qPCR, 5 and 10 µl of input DNA samples from panels 1, 2 and 3 shall be tested!**

6. ***Spiking of salad***

Perform the "spiking" and the "oocyst recovery step" as soon as possible (optimally 24-48h from sample arrival). The sediment pellet can be stored (strongly suggested) at -20°C before proceeding with DNA extraction.

Before proceeding with spiking, the matrix should be tested to ensure it is *T. gondii*-free by applying the SOP on, at least, 30 g of salad. If not *T. gondii* free, salad control (unspiked) should be processed and tested at the same time as spiked samples.

7. ***Spiking procedure***

1) Prepare 5 filter bags (or 6 if an unspiked control is run in parallel) on a stand (or in a beaker).

2) In each filter-bag weigh out approx. 30 g of the leafy salad. Keep the bags propped upright to minimize the risk of leakage or splashing during oocyst spiking.

3) Spin-down each tube of the Panel 1 (few seconds at max speed) to ensure all drops and oocysts are collected from the tube wall.

4) Gently pipette up and down several times to mix the suspension. Do not vortex, to avoid the suspension spreading on the tube wall.

5) Dispense the entire suspension (approximately 100 µl) in 10 droplets (≈10 µl each) distributed across different areas of the sample (aim for the center of leaves to ensure that the spike suspension does not run off).

6) Leave to dry at room temperature for 2-3 h with the filter-bag open.

7) Close the bag by folding the edge, secure with adhesive tape and place at 4°C overnight

8) The day after, start processing the samples (SOP paragraph 7.2.1).

8. ***Criteria for result evaluation***

Results evaluation is qualitative, the participant is required to correctly identify RT items as positive or negative. To provide further data on the procedure, participants should also report Ct values obtained (mean values with standard deviation and number of positive replicas/total replicas).

9. ***Report***

Within 10 working days after the due date to submit results, an individual report will be provided to each participant including the following information: i) expected result (positive/negative); ii) observed result (positive/negative); iii) final evaluation and iv) comments based on the laboratory performance. The ISS will also compile a Final Report including results obtained by all participants. The Final Report will be discussed with all participants during an ad hoc online meeting and will be part of a scientific publication.

Supplementary Methods

Preparation of oocyst aliquots for spiking and salads leaves and salad sediment spiking procedure

To prepare oocyst aliquots for spiking, oocysts were initially diluted to $1x10^5$/ml in sterile PBS and counted (in duplicate by two operators) in glasstick chamber slides, then further diluted in the appropriate volume of PBS (50 µl for pellet spiking and 100 µl for leaf spiking). After every 3 spikes, an aliquot of oocysts (50 or 100 µl) was spotted onto slides, air dried, mounted using a mounting medium and counted under a fluorescent microscope (UV light) and the average oocyst count was reported. Salad spiking was conducted as follows: ≈30 g of mixed salad was transferred into a homogenization filter bag and, for each 100 µl aliquot, 10 drops of 10 µl were randomly spotted onto the leaves. Drops were left to dry at room temperature and bags were then incubated overnight at 4°C before proceeding with the test. Salad sediments (approximately 250 µl) were mixed with 50 µl of oocyst suspension and stored at -20°C until use.

Selection of ready-to-eat salad mixes

Baby leaves are marketed as a mixture of fresh immature young leaves with attractive colors and shapes that are flavourful and highly nutritious; cut salads are marketed as good quality fresh-cut products harvested at optimal maturity stage [1,2]. To ensure representativeness and to increase comparability of the two categories of salad mixes, the ingredient compositions of the salads included in the multi-center survey were selected based on several considerations. The primary focus of the survey was on fresh produce that may be at higher risk of contamination with *T. gondii* oocysts and literature review data were used to support the selection [3] (Figure 1A). Since the risk of infection with *T. gondii* oocysts is directly linked to food consumption, information was collected on the most common RTE salad mixes that could be purchased by consumers in stores (supermarkets) in the ten European countries involved in the European-wide study. Information included: supermarket name, salad brand, salad name, type of cultivation (organic [Y/N]), salad origin (by country), name of the producer, name of the packaging company, location of the packaging facility, salad composition, and package barcode. Data were collected for 165 RTE salad mixes (including three products from organic cultivation), representing 34 brands, in 27 supermarkets located in eleven cities: Brno (Czech Republic), Copenhagen (Denmark), Paris (France), Berlin (Germany), Rome (Italy), Pulawy (Poland), Lisbon (Portugal), Oslo (Norway), Madrid (Spain), and Basingstoke/Guildford (United Kingdom). To avoid potential confounders, only salad mixes including root vegetables (e.g. carrots) or other ingredients (e.g. seeds, herbs) were excluded as only green leaves were used for validation of the test procedure. The composition of each salad was reported and listed to define the commonest mixes available in the majority of countries. Data from other sources [7], such as ongoing surveys of pathogens in leafy greens were also taken into consideration when deciding on product selection. Based on this information, salad mixes containing three to four of the following ingredients were considered suitable for sampling in the multicentre study: baby spinach (*Spinacia oleracea*), arugula (*Eruca vesicaria*), baby lamb's lettuce (*Valerianella locusta*), baby red and green lettuce (*Lactuca sativa*) for baby leaf mixes (BABY LEAVES); frisée (curly escarole) lettuce (*Chicorium endivia*), radicchio (red) (*Cichorium intybus var. foliosum*), endive (*Cichorium intybus*), any green lettuce (*Lactuca sativa*) (e.g. iceberg, romain, etc.) for cut salad mixes (CUT SALADS). If salad mixes were not available, salad monotypes for each of the two categories (baby leaf monotype or cut leaf monotype) were sampled instead.

References

1. Gil MI, Garrido Y. The impact of light on modified atmosphere storage and quality of fresh produce. In Controlled and Modified Atmospheres for Fresh and Fresh-Cut Produce, 1st ed.; Gil, M.I., Beaudry, R., Eds.; Academic Press: Cambridge, MA, USA, 2020. p. 167-184. https://doi.org/ 10.1016/B978-0-12-804599-2.00010-7

2. Tudela, JA; Gil, MI Leafy vegetables: Fresh-cut lettuce. In Controlled and Modified Atmospheres for Fresh and Fresh-Cut Produce, 1st ed.; Gil, M.I., Beaudry, R., Eds.; Academic Press: Cambridge, MA, USA, 2020. p. 545–550. https://doi.org/10.1016/B978-0-12-804599-2.00044-2.

3. López-Ureña NM, Chaudhry U, Calero-Bernal R, Cano-Alsua S, Messina D, Evangelista F, et al. Contamination of Soil, Water, Fresh Produce, and Bivalve Mollusks with *Toxoplasma gondii* Oocysts: A Systematic Review. Microorganisms. 2022;10(3):517. https://doi.org/10.3390/microorganisms10030517. PMID: 35336093.

Supplementary Tables and Figures

Supplemental TABLE S1 Summary of precision of prevalence estimates with a sample size of 400.

Sample size	Design effect[a]	Precision of prevalence estimate (at 1%)[b]	Precision of prevalence estimate (at 20%)[b]
400	1	1.0%	4%
400	2	1.4%	6%
400	3	1.7%	7%
400	4	2.0%	8%

[a] Takes into account intra-cluster correlation at country level but cannot be estimated without data;
[b] Considering a confidence level of 95%

Supplemental TABLE S2 List of reagents, equipment and settings validated during interlaboratory validation and later applied by laboratories during the ready-to-eat salad survey.

Country	Filter bags	Paddle Homogenizer		Beat-beating Homogenizer		DNA extraction kit	real-time PCR MM	real-time PCR platform	
			settings		settings				Settings
Czechia	Side filter bag (VWR, #129-0733)	BagMixer (interscience SW400)	2 x 30 sec at “normal speed”	Precellys 24 (Bertin Instruments)	6400 rpm/60 seconds at 6400 rpm/	DNeasy PowerSoil Kit (Qiagen)	SensiFAST™ Probe Lo-ROX Kit (Bioline)	LightCycler 480 (Roche)	As in SOP
Germany	Side filter bag (VWR, #129-0733)	BagMixer (interscience SW400)	2 x 30 sec at setting 1: 4 beats/s = 240 rpm	FastPrep24 (MPBio)	2 x 40 sec at amplitude of 6.0	FastDNA Spin Kit for Soil (MP)	SensiFAST™ Probe Lo-ROX Kit (Bioline);	7500 Fast Real-time PCR System (Applied Biosystems)	As in SOP
Denmark	Side filter bag (VWR, #129-0733)	Star blender LB 400 (VWR Int.)	2 x 30 sec at maximum speed	Qiagen TissueLyser 30Hz	2 x 5 min at 25 Hz	DNeasy PowerSoil Kit (Qiagen)	PerfeCTa qPCR ToughMix (QuantaBio)	QuantStudio™ 5 Real-Time PCR System for Human Identification, 96-well, 0.2 mL, desktop	activation 95°C 2 min; 45 cycles at 95 15 sec R rate 2.66; 60°C 40 sec R rate 2.12
Poland	Blender bag with Lateral filter (VWR, #129-0733)	Star Blender Digital (VWR Int.)	2 x 30 sec at 300 rpm	FastPrep24 (MPBio)	2 x 40 sec at amplitude of 6.0	FastDNA Spin Kit for Soil (MP)	SensiFast^{TM} Probe Lo-ROX Kit (Bioline)	Bio-rad CFX96	As in SOP
UK	Blender bag with Lateral filter (VWR, #129-0733)	Star Blender Digital (VWR Int.)	2 x 30 sec at 300 rpm	Qiagen TissueLyser LT	3 x 3 min at 50 Hz	DNeasy PowerSoil Kit (Qiagen)	SensiFAST^{TM} Probe no ROX (Bioline)	Bio-rad CFX96	As in SOP
Spain	Side filter bag (VWR, #129-0733)	Stomacher, Nr. 2188/480 (IUL Instrument)	2 x 30 sec at maximum speed	Biospec Mini-Beadbeater 16	Maximum speed, no speed regulator (1500 rpm)/ 60 sec	FastDNA Spin Kit for Soil (MP)	SensiFAST™ Probe Lo-ROX Kit (Cat. BIO-84005)	7500 Fast Real- time PCR System (Applied Biosystems)	As in SOP
Norway	Blender bag with Lateral filter (VWR 129-0733)	Stomacher Bag Mixer 400 CC (Interscience)	2 x 30 sec at speed 2 (out of 4)	Bead Beater Precellys Evolution (Bertin Technologies)	2 x 40 sec at amplitude of 6.0	FastDNA Spin Kit for Soil (MP)	Brilliant Multiplex QPCR Master Mix #600553 (Agilent)	Bio-rad CFX96	As in SOP
France	Full filter bags (Seward Stomacher 400C Sterile Strainer)	Smasher (AES Laboratoire)	2 x 30 sec at speed “Rapide”	Precellys 24 (Bertin Instruments)	6400 rpm/60 seconds	DNeasy PowerSoil Kit (Qiagen)	Premix Ex Taq no ROX (Takara)	LighCycler 480 II (Roche)	As in SOP
Italy	Side filter bag (VWR, #129-0733)	Star Blender Digital (VWR Int.)	2 x 30 sec at 300 rpm	FastPrep24 (MPBio)	2 x 40 sec at amplitude of 6.0	FastDNA Spin Kit for Soil (MP)	LightCycler Multiplex DNA Master no ROX 5XMultiplex PCR Kits no ROX (Roche)	LightCycler 96 (Roche)	As in SOP
Portugal	Side filter bag (VWR, #129-0733)	Stomacher 80 (Seward)	2 x 30 sec at medium speed	N/A	N/A	N/A	N/A	N/A	N/A

Supplemental TABLE S3 Performance of triplex real-time PCR using plasmid DNA and different DNA polymerases and real-time PCR platforms

qPCR thermocycler	Taq polymerase[a]	Triplex_10000[b]			Triplex_1000[b]			Triplex_100[b]			Triplex_10[b]		
		Mean Ct ± SD (N=6)											
		IAC[c]	B1	529RE	IAC[c]	B1	529RE	IAC[c]	B1	529RE	IAC[c]	B1	529RE
LightCycler 96 (Roche)	SensiFAST Probe (Bioline)	23.00 ± 0.46	18.02 ± 0.08	16.58 ± 0.38	22.86 ± 0.40	22.40 ± 0.93	21.37 ± 0.78	22.67 ± 0.87	26.04 ± 1.12	25.02 ± 0.95	22.91 ± 0.83	29.02 ± 1.04	28.26 ± 0.95
	QuantiFast Multiplex PCR (Qiagen)	22.93 ± 0.60	18.79 ± 0.17	17.80 ± 0.57	23.03 ± 0.29	22.86 ± 0.93	22.32 ± 0.76	23.00 ± 0.65	26.17 ± 0.91	25.80 ± 0.73	23.08 ± 0.86	29.56 ± 0.82	29.5 0± 0.78
LightCycler 480 (Roche)	LightCycler 480 Probes Master (Roche)	29.30 ± 1.01	25.54 ± 1.80	25.83 ± 0.29	29.68 ± 0.94	28.83 ± 1.56	29.16 ± 0.22	29.78 ± 0.78	32.34 ± 1.75	32.37 ± 0.38	29.75 ± 0.70	35.15 ± 1.93	35.19 ± 0.27
	Luna Universal Probe qPCR Master Mix (NEB)	29.22 ± 0.96	25.58 ± 0.15	24.09 ± 0.15	29.55 ± 0.86	28.57 ± 0.13	27.07 ± 0.05	30.33 ± 0.62	32.03 ± 0.28	30.14 ± 0.07	30.15 ± 0.81	34.57 ± 0.53	33.61 ± 0.60

[a] All master mixes are without ROX dye
[b] copy number of each target gene (B1 and 529RE)
[c] IAC copy number for each reaction is 120.

Supplemental TABLE S4 Performance of triplex real-time PCR using different DNA polymerases and real-time PCR platforms on genomic DNA extracted from purified *T. gondii* oocysts.

DNA from oocysts (ng)	Oocyst equivalent (number)[a]	Gene Name	qPCR Platform											
			LightCycler 96 (Roche)						LightCycler 480 (Roche)					
			**Taq Polymerase**											
			SensiFAST Probe (Bioline)			QuantiFast Multiplex PCR (Qiagen)			LightCycler 480 Probes Master (Roche)			Luna Universal Probe qPCR Master Mix (NEB)		
			**Mean Ct**	**SD**	**POD[b]**	**Mean Ct**	**SD**	**POD**	**Mean Ct**	**SD**	**POD**	**Mean Ct**	**SD**	**POD**
560	1000	IAC	23.7	1.46	100% (12/12)	24.03	2.01	100% (9/9)	30.55	1.85	100% (9/9)	28.3	1.99	100% (9/9)
		B1	19.23	0.49	100% (12/12)	19.44	0.27	100% (9/9)	21.6	2.03	100% (9/9)	22.43	4.25	100% (9/9)
		529	14.09	0.9	100% (12/12)	15.14	0.38	100% (9/9)	19.47	1.32	100% (9/9)	18.34	4.81	100% (9/9)
56	100	IAC	23.68	1.69	100% (12/12)	24.27	1.88	100% (9/9)	31.09	3.78	100% (9/9)	29.44	1.8	100% (9/9)
		B1	22.36	0.24	100% (12/12)	22.85	0.23	100% (9/9)	24.5	0.99	100% (9/9)	26.22	2.64	100% (9/9)
		529	17.29	0.43	100% (12/12)	19.03	0.31	100% (9/9)	22.45	1.09	100% (9/9)	22.56	3.75	100% (9/9)
5.6	10	IAC	23.45	1.75	100% (12/12)	24.31	2.13	100% (9/9)	29.59	1.58	100% (9/9)	29.08	0.8	100% (9/9)
		B1	25.58	0.44	100% (12/12)	26.26	0.75	100% (9/9)	27.73	1.04	100% (9/9)	29.34	1.78	100% (9/9)
		529	20.85	0.64	100% (12/12)	22.8	0.86	100% (9/9)	25.82	1.17	100% (9/9)	25.85	3.92	100% (9/9)
0.56	1	IAC	23.56	1.46	100% (12/12)	24.01	2.21	100% (9/9)	29.96	1.26	100% (9/9)	29.56	1.14	100% (9/9)
		B1	28.72	0.6	100% (12/12)	29.21	0.85	100% (9/9)	30.79	1.03	100% (9/9)	31.99	1.34	100% (9/9)
		529	24.16	0.9	100% (12/12)	25.59	0.84	100% (9/9)	29.13	1.34	100% (9/9)	27.8	1.69	100% (9/9)
0.056	0.1	IAC	23.56	1.37	100% (12/12)	23.91	2.17	100% (9/9)	30.14	1.24	100% (9/9)	29.63	1.55	100% (9/9)
		B1	30.78	0.91	100% (12/12)	30.89	1	100% (9/9)	33.66	0.94	100% (9/9)	34.88	2.42	67% (6/9)
		529	26.31	0.98	100% (12/12)	27.19	0.73	100% (9/9)	32.93	2.3	100% (9/9)	30.28	1.49	100% (9/9)
0.0056	0.01	IAC	24.54	1.06	100% (9/9)	26.2	0.3	100% (6/6)	-	-	-	-	-	-
		B1	35.74	2.44	22% (2/9)	40.84	1.1	0% (0/6)	-	-	-	-	-	-
		529	33.25	1.83	100% (9/9)	28.3	22.67	33% (2/6)	-	-	-	-	-	-
0.00056	0.001	IAC	24.54	1.09	100% (9/9)	25.97	0.33	100% (6/6)	-	-	-	-	-	-
		B1	0.00	0.00	0% (0/9)	0.00	0.00	0% (0/6)	-	-	-	-	-	-
		529	0.00	0.00	0% (0/9)	0.00	0.00	0% (0/6)	-	-	-	-	-	-

a) Oocyst equivalent number was calculated based on DNA concentration considering 70 fg of DNA/T. gondii haploid genome and 8 haploid genomes/T. gondii oocyst (560 fg of DNA/*T. gondii* oocyst). DNA extraction was performed with the FastDNA™ Spin Kit for Soil (MPBio). b) POD (%): Probability of Detection (number of positive real-time PCR replicates/total number of real-time PCR replicates) x 100. POD values <100% are highlighted in grey.

Supplemental TABLE S5 Performance of triplex real-time PCR using DNA extracted from salad pellets spiked with *T. gondii* oocysts and different DNA polymerases and real-time PCR platforms.

Spiking level[a] (N replicates)[b]	Theoretical oocyst N/reaction	Gene Name	DNA Extraction Kit	qPCR Platform: LightCycler 96 (Roche); DNA Polymerase: Bioline SensiFAST Probe				qPCR Platform: LightCycler 96 (Roche); DNA Polymerase: Qiagen QuantiFast Multiplex PCR				DNA Extraction Kit	qPCR Platform: LightCycler 480 (Roche); DNA Polymerase: LightCycler 480 Probes Master (Roche)				qPCR Platform: LightCycler 480 (Roche); DNA Polymerase: Luna Universal Probe qPCR Master Mix (NEB)			
				**Cq Mean**	**Cq Error**	**POD[c]**	**SE[d]**	**Cq Mean**	**Cq Error**	**POD**	**SE**		**Cq Mean**	**Cq Error**	**POD**	**SE**	**Cq Mean**	**Cq Error**	**POD**	**SE**
0 (N=6)	0	IAC	FastDNA™ Spin Kit for Soil (MPBio)	22.97	0.54	100% (12/12)	NA	23.11	0.57	100% (12/12)	NA	DNeasy PowerSoil Kit (Qiagen)	31.07	0.28	100% (12/12)	NA	28.40	0.39	100% (12/12)	NA
		B1		0.00	0.00	0% (0/12)	NA	0.00	0.00	0% (0/12)	NA		0.00	0.00	0% (0/12)	NA	0.00	0.00	0% (0/12)	NA
		529		0.00	0.00	0% (0/12)	NA	0.00	0.00	0% (0/12)	NA		0.00	0.00	0% (0/12)	NA	0.00	0.00	0% (0/12)	NA
100 (N=6)	10	IAC		22.72	0.88	100% (18/18)	NA	22.11	0.10	100% (9/9)	NA		30.75	1.23	100% (24/24)	NA	30.10	0.40	100% (18/18)	NA
		B1		28.00	1.05	100% (18/18)	100% (6/6)	29.16	0.38	100% (9/9)	100% (3/3)		32.83	1.66	100% (24/24)	100% (6/6)	31.70	0.42	100% (18/18)	100% (6/6)
		529		23.65	0.63	100% (18/18)	100% (6/6)	25.62	0.27	100% (9/9)	100% (3/3)		29.45	0.68	100% (24/24)	100% (6/6)	26.94	0.63	100% (18/18)	100% (6/6)
50 (N=6)	5	IAC		22.68	0.82	100% (18/18)	NA	22.19	0.30	100% (9/9)	NA		30.54	1.55	100% (24/24)	NA	30.23	0.44	100% (18/18)	NA
		B1		29.12	1.07	100% (18/18)	100% (6/6)	30.62	0.58	100% (9/9)	100% (3/3)		34.11	1.86	100% (24/24)	100% (6/6)	33.30	1.20	100% (18/18)	100% (6/6)
		529		24.99	0.64	100% (18/18)	100% (6/6)	26.72	0.65	100% (9/9)	100% (3/3)		30.87	0.92	100% (24/24)	100% (6/6)	28.28	0.79	100% (18/18)	100% (6/6)
10 (N=6)	1	IAC		22.86	0.75	100% (18/18)	NA	22.05	0.10	100% (9/9)	NA		30.57	1.19	100% (24/24)	NA	30.43	0.50	100% (18/18)	NA
		B1		31.96	1.44	94% (17/18)	100% (6/6)	33.47	2.27	100% (9/9)	100% (3/3)		35.66	2.22	100% (24/24)	100% (6/6)	35.73	1.75	61% (11/18)	67% (4/6)
		529		28.51	2.26	100% (18/18)	100% (6/6)	30.73	1.49	100% (9/9)	100% (3/3)		33.19	1.59	100% (24/24)	100% (6/6)	30.64	1.48	89% (16/18)	100% (6/6)
100 (N=3)	10	IAC	QIAamp Fast DNA Stool Mini (Qiagen)	22.21	0.40	100% (9/9)	NA	22.11	0.11	100% (9/9)	NA	QIAamp Fast DNA Stool Mini (Qiagen)	29.17	0.31	100% (9/9)	100% (3/3)	30.91	0.21	100% (9/9)	NA
		B1		36.16	1.53	67% (6/9)	67% (2/3)	36.75	0.19	100% (9/9)	100% (3/3)		34.94	1.12	100% (9/9)	100% (3/3)	34.53	0.60	100% (9/9)	100% (3/3)
		529		32.12	2.46	100% (9/9)	100% (3/3)	34.58	2.46	100% (9/9)	100% (3/3)		33.16	0.71	100% (9/9)	100% (3/3)	29.82	0.69	100% (9/9)	100% (3/3)
50 (N=3)	5	IAC		22.18	0.17	100% (9/9)	NA	22.23	0.27	100% (9/9)	NA		29.40	0.24	100% (9/9)	NA	30.90	0.14	100% (9/9)	NA
		B1		36.89	1.25	45% (4/9)	67% (2/3)	35.32	1.10	78% (7/9)	100% (3/3)		35.01	0.98	33% (3/9)	33% (1/3)	35.44	1.20	22% (2/9)	33% (1/3)
		529		34.32	1.19	89% (8/9)	100% (3/3)	35.36	0.81	100% (9/9)	100% (3/3)		33.88	0.66	100% (9/9)	100% (3/3)	30.37	0.58	89% (8/9)	100% (3/3)
10 (N=3)	1	IAC		22.18	0.32	100% (9/9)	NA	22.10	0.05	100% (9/9)	NA		29.35	0.26	100% (9/9)	NA	31.02	0.14	100% (9/9)	NA
		B1		36.44	0.00	11% (1/9)	33% (1/3)	0.00	0.00	0% (0/9)	0% (0/3)		36.90	0.33	22% (2/9)	33% (1/3)	38.21	0.00	22% (2/9)	33% (1/3)
		529		33.94	0.20	33% (3/9)	67% (2/3)	35.77	1.06	45% (4/9)	67% (2/3)		37.03	1.52	56% (5/9)	67% (2/3)	32.77	1.73	33% (3/9)	33% (1/3)

SD: standard deviation; NA: not applicable; a) Spiking level is the number of oocyst/30 g of RTE salad. b) Number of replicates tested for each experimental condition; c) POD: Probability of Detection (number of positive qPCR replicates/total number of qPCR replicates) x 100; d) SE: Sensitivity (number of positive samples/total spiked samples). A sample was considered positive if at least one real-time PCR replicate produced a positive result with Ct ≤ 3

Supplemental TABLE S6 Number of positive samples by packaging facility

Country	Packaging facility	Total number	Number real-time PCR positive	Percent real-time PCR positive
Italy	1	1	0	0.00
	2	41	0	0.00
	3	7	1	14.29
	4	1	0	0.00
	5	66	1	1.52
	6	23	0	0.00
	7	25	0	0.00
	8	80	3	3.75
	9	70	1	1.43
	10	2	0	0.00
	11	1	0	0.00
France	12	15	0	0.00
	13	98	7	7.14
	14	3	0	0.00
	15	132	9	6.82
	16	38	1	2.63
	17	3	0	0.00
	18	103	7	6.80
Denmark	19	2	0	0.00
	20	166	4	2.41
	21	20	0	0.00
	22	104	7	6.73
	23	28	1	3.57
Germany	12	103	3	0.00
	15	3	0	2.91
	24	165	5	3.03
	25	5	0	0.00
	26	94	1	1.06

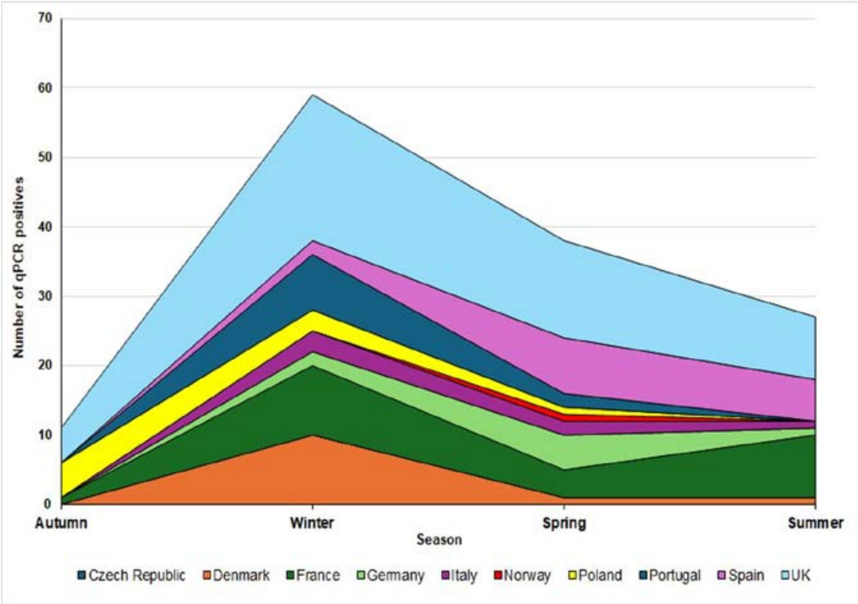

Supplemental FIGURE S1 Number of positive samples to *Toxoplasma gondii* by real-time PCR per season per country.

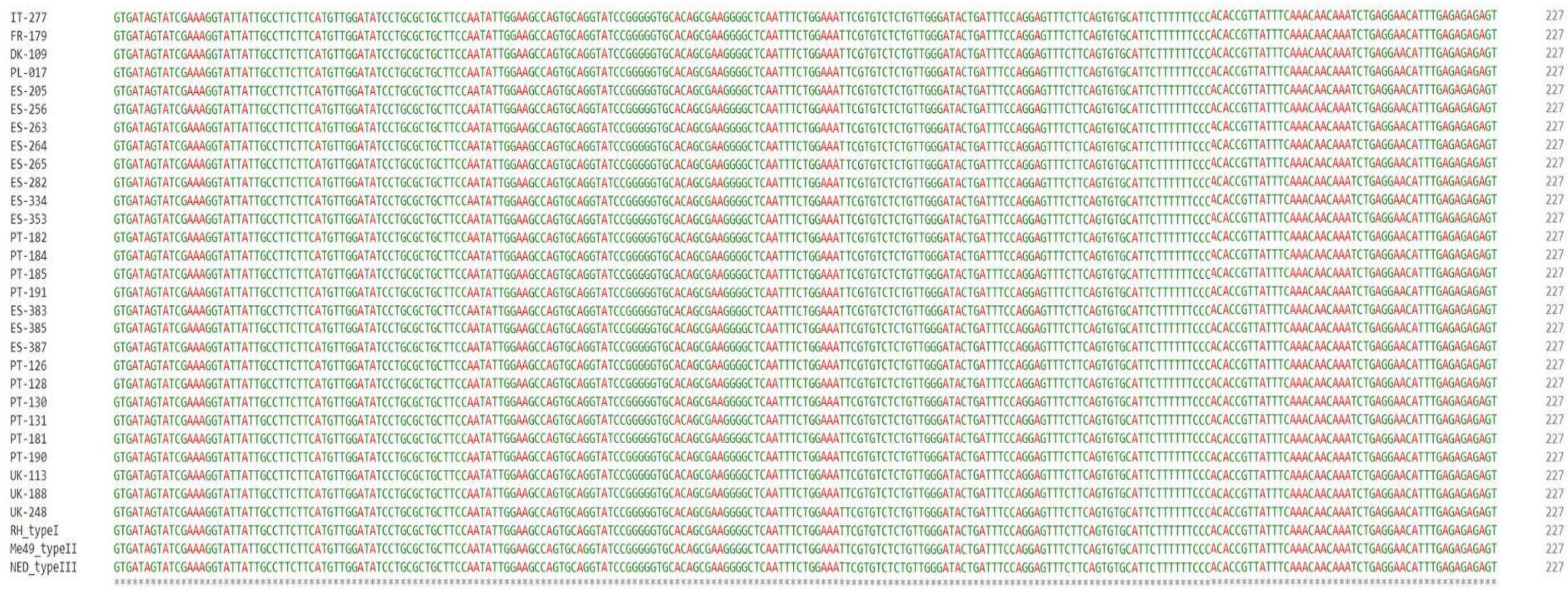

Supplemental FIGURE S2 Multiple sequence alignment of ITS-1 amplicons from DNA extracted from RTE salad samples with *Toxoplasma gondii* ITS-1 sequences obtained herein from reference strains TgRH, TgMe49 and TgNED.
